# Genomic data define species delimitation in Liberica coffee with implications for crop development and conservation

**DOI:** 10.1038/s41477-025-02073-y

**Published:** 2025-08-08

**Authors:** A. P. Davis, A. Shepherd-Clowes, M. Cheek, J. Moat, D. Wei Luo, C. Kiwuka, J. Kalema, B. Tchiengué, J. Viruel

**Affiliations:** 1 Royal Botanic Gardens, Kew, Richmond, UK; 2 National Agricultural Research Organization, Entebbe, Uganda; 3 Makerere University Herbarium, Kampala, Uganda; 4 IRAD–National Herbarium of Cameroon, Yaoundé, Cameroon

**Keywords:** Plant evolution, Plant sciences

## Abstract

Safeguarding the long-term future of the global coffee supply chain represents a major challenge, particularly in an era of accelerated climate change. Of particular concern are the millions of smallholder farmers across the tropical belt who rely on coffee as their major source of income. The world’s coffee farmers, and thus the global coffee supply chain, rely on two species: Arabica (*Coffea arabica*) and robusta (*Coffea canephora*)^1^. A third species, *Coffea liberica*, including Liberica coffee (*C.* *liberica* var. *liberica*) and excelsa coffee (*C.* *liberica* var. *dewevrei*), represents a minor share of global production, although the cultivation of this species is steadily increasing owing to climate challenges affecting Arabica and robusta, coupled with an increasing market demand^2^. In Southeast Asia, Liberica consumption has continued since its introduction in the late-nineteenth century and is now witnessing a renaissance, particularly in Malaysia, Indonesia and Fiji. In Uganda, South Sudan and Guinea, attention is focused on excelsa owing to its ability to grow and produce commercially viable crops under higher temperatures and extended periods of low rainfall compared with robusta^2,3^. Excelsa production is also increasing in India in response to worsening climate conditions for Arabica and robusta, and in Vietnam and Indonesia to supplement robusta and diversify coffee production. Here we investigate species delimitation in *C.* *liberica* using genomic data in combination with morphology and geographical distribution, to understand the implications for coffee crop improvement and the conservation of coffee genetic resources.

## Abstract (web summary)

Genomic and other datasets support the division of *Coffea liberica* into three species: *C.* *liberica*, *C.* *dewevrei* (excelsa) and *C.* *klainei*. This has implications for crop use and development, as well as species conservation.

## Main

Our limited understanding of the diversity and trait partitioning within *Coffea liberica* constrains its utilization and development. The taxonomic delimitation and identification of *C.* *liberica* continues to confound researchers and coffee value-chain stakeholders, with inconsistent and confusing use of scientific and vernacular names in published research, agriculture and the media. The current consensus of taxonomic and systematic study^4–7^ is that *C.* *liberica* is a single species, divided into two botanical varieties: var. *liberica* and var. *dewevrei*^8^. While this classification is generally accepted, it is also argued that it does not fully account for the morphological^9,10^, and potential molecular variation^4,11,12^, within the species, and thus requires further critical study^2,3^. An alternative viewpoint is that *C.* *liberica* represents a single species with no infraspecific taxa^13^. Simply put, what is Liberica coffee and does it represent one, or more, species?

## A revised species delimitation for Liberica

Here, we demonstrate congruence across genomic, morphological and spatial analyses, and elucidate distinct evolutionary lineages^14^, supporting the division of *C.* *liberica* into three distinct species: *C.* *liberica* (Liberica), *C.* *dewevrei* (excelsa) and *C.* *klainei*, following the rules of nomenclatural priority^15^. *C.* *klainei* is a poorly known species, previously considered to represent a synonym of *C.* *liberica*^5,16^. With *C.* *dewevrei* and *C.* *klainei* reinstated, the total number of known *Coffea* (coffee) species increases from 131 (ref. ^8^) to 133. Cameroon gains two species (now 18 species in total) and becomes the African country with the highest number of indigenous species, followed by Tanzania (17 species) and second only to Madagascar with 67 species^8^.

## Phylogenomic analyses

**Fig. 1:**
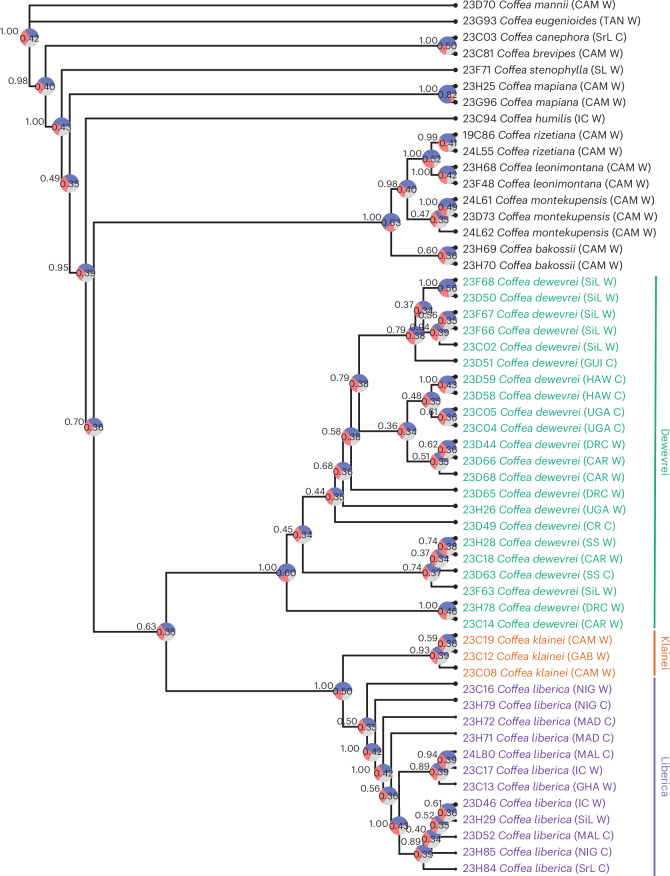
Inferred relationships for *C.* *liberica* (Liberica), *C.* *dewevrei* (excelsa), *C.* *klainei* and allied species based on sequencing of Angiosperms353 nuclear genes. An ASTRAL tree. The pie charts show the QSs informing on the agreement between genes with LPP scores. For BS values, see Supplementary Fig. 1. C, cultivated (accessions from farms or germplasm collections); W, wild (accessions from natural (indigenous) populations). Accession information and country codes are provided in Supplementary Table 1.

We used the Angiosperms353 target capture kit^17,18^ to elucidate phylogenomic relationships within *C.* *liberica* sensu lato and related species. This genomic tool resolves relationships at various levels of taxonomic hierarchy in flowering plants^19,20^, including those at the population scale^21^. We sequenced 353 nuclear genes from 55 accessions (Supplementary Table 1). Across all accessions, we recovered 68.7–91.2% (mean 86.5%) of the 353 genes, representing 196,392–262,188 (mean 248,453) bp per sample recovered across the sample set. The mean number of gene sequences missing per species in the final alignments was 0.17, ranging from 1–10. Overall, there were 3.17% missing data in the alignments. These statistics exclude *C.* *magnistipula*, for which only small percentage of the 353 genes were recovered (see below). Inferred relationships for *C.* *liberica* sensu lato and allied species are shown in Fig. 1, based on a species tree obtained from a multispecies-coalescent ASTRAL-III analysis. Pie charts are placed at the nodes, showing the quartet scores (QSs) (maximum of 1.00), informing on the agreement between genes in the most likely phylogenomic topologies reconstructed, and the local posterior probability (LPP) scores (maximum of 1.00). The bootstrap (BS) values are provided in a supermatrix tree in Supplementary Fig. 1.

ASTRAL-III analysis grouped all *C.* *liberica* sensu lato accessions into a single clade (QS 0.63, LPP 0.36, BS 99), which subdivides into three monophyletic clades: *C.* *liberica* (QS 0.35, LPP 0.50, BS 100), *C.* *klainei* (QS 0.39, LPP 0.93, BS 99) and *C.* *dewevrei* (QS 0.60, LPP 1.00, BS 100). *C.* *liberica* and *C.* *klainei* were retrieved as a clade (QS 0.50, LPP 1.00, BS 60), sister to the *C.* *dewevrei* clade. These results support the phylogenomic distinction of the three species. The relatively low QS and LPP values are addressed in the single-nucleotide polymorphism (SNP) analyses section below. The remaining *Coffea* species fall outside the aforementioned clades, including a sister clade of five endemic species from Cameroon (*C.* *bakossii*, *C.* *rizetiana*, *C.* *leonimontana*, *C.* *mapiana* and *C.* *montekupensis*), *C.* *humilis* (indigenous to Guinea, Sierra Leone, Liberia and Ivory Coast) and *C.* *magnistipula* (indigenous to Cameroon and Gabon). The outgroups (*C.* *stenophylla*, *C.* *brevipes*, *C.* *eugenioides* and *C.* *mannii*) fall into positions that are congruent with published phylogenetic analyses of *Coffea*^6,12,22,23^. We were only able to recover a small percentage (19.7%) of 353 genes for the sample of *C.* *magnistipula*, and so this species was not included in the ASTRAL tree (Fig. 1). A separate ASTRAL-III analysis shows that this species falls between *C.* *mapiana* and *C.* *humilis* (Supplementary Figs. 1 and 2), as anticipated based on morphological, geographical and ecological similarities with *C.* *mapiana*^24^. No direct comparison can be made with previous molecular phylogenetic studies for *C.* *liberica* relatives^6,22,23,25^ owing to either their limited taxon sampling or low levels of sequence data or both.

## SNP analyses

**Fig. 2:**
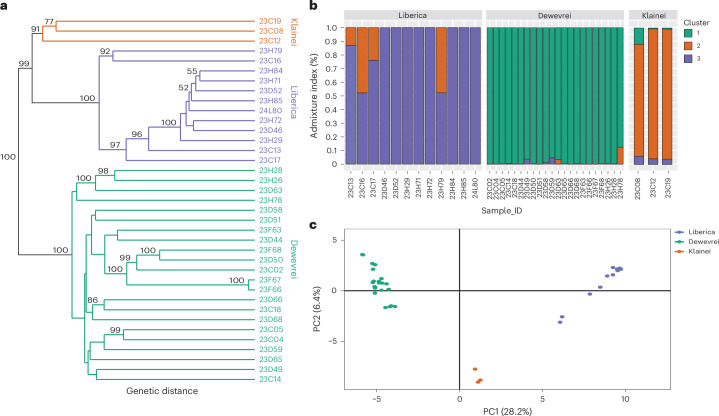
Genetic structure and relationships between and within *C.* *liberica* (Liberica), *C.* *dewevrei* (excelsa) and *C.* *klainei*, based on 2,240 exon region SNPs. **a**, A genetic distance phylogenetic tree reconstructed with pairwise genetic distances (the proportion of loci that are different). The figures above branches indicate the BS values (BS values <50 not shown). **b**, STRUCTURE^26^ analysis with the *K* value set to *K* = 3 to match the phylogenomic analysis (Fig. 1), genetic distance tree (**a**), PCoA analyses (**c**) and geographical distribution (Fig. 3), representing three clusters (*C. liberica*, *C. dewevrei* and *C. klainei*; see the main text and Supplementary Fig. 3 for alternative *K* values and details). **c**, PCoA analysis. PC1 (28.2% variance) separates the three species, with *C.* *klainei* intermediate to *C.* *liberica* and *C.* *dewevrei*. Acc*e*ssion information is provided in Supplementary Table 1.

**Fig. 3:**
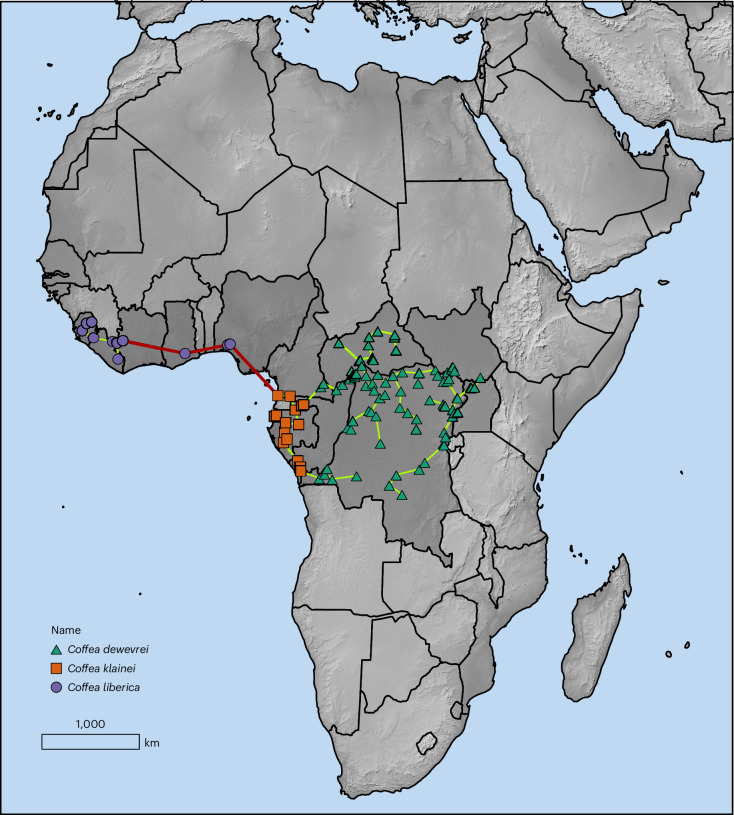
Distribution map for indigenous (wild) *C.* *liberica* (Liberica), *C.* *dewevrei* (excelsa) and *C.* *klainei.* The colours of the symbols are matched to the genetic distance phylogenetic tree (Fig. 2a). Linked symbols represent a Rapoport’s mean propinquity assessment^29^ using a barrier distance of 500 km, which reveals a robust population separation between *C.* *liberica* versus *C.* *dewevrei* and *C.* *klainei*, but not between *C.* *dewevrei* and *C.* *klainei*.

To investigate the genetic structure and relationships between and within *C.* *liberica*, *C.* *klainei* and *C.* *dewevrei*, we utilized 2,240 SNPs from the exon regions^21^ of 37 accessions (Supplementary Table 1). A genetic distance phylogenetic tree reconstructed with pairwise genetic distances supports the monophyly of the three species (BSs of 100, 91 and 100, respectively; Fig. 2a). *C.* *liberica* and *C.* *klainei* form a clade (BS 99) sister to *C.* *dewevrei*, consistent with the relationship recovered in the phylogenomic analysis (Fig. 1). Samples 23C16 (Nigeria) and 23H79 (Ivory Coast) form a clade (BS 92) sister to other *C.* *liberica* samples. On principal coordinate analysis (PCoA) (Fig. 2c) PC1 (28.2% variance) clearly separates the three species, with *C.* *klainei* positioned between *C.* *liberica* and *C.* *dewevrei*, with PC2 (6.4%) showing two outliers (23C16 and 23H79) for *C.* *liberica*. On the STRUCTURE^26^ analysis (Fig. 2b) we set the *K* value to *K* = 3, to match the phylogenomic analysis (Fig. 1), genetic distance tree (Fig. 2a), PCoA analyses (Fig. 2c) and geographical distribution (Fig. 3), representing three clusters (*C.* *liberica*, *C.* *dewevrei* and *C.* *klainei*). For *K* = 3, in *C.* *liberica* there is admixture from the *C.* *klainei* genetic group: 47.8% for 23C16 (Nigeria), 47.7% for 23H79 (Ivory Coast), 13% for 23C13 (Ghana) and 24% for 23C17 (Ivory Coast) (Fig. 2b). In *K* = 4 and *K* = 5, these four admixtures are not from *C.* *klainei* (Supplementary Fig. 3). These outliers require further investigation. *K* = 2 was identified as the most likely number of *K* genetic clusters^26–28^ (Δ*K* value of 3,605.7, compared with <Δ*K* of 37.54 for the remaining *K* values assessed; Supplementary Fig. 3). *K* = 2 was rejected on the grounds that it underrepresented population genetic structure for our study group^29^ and was incongruent with the known biological information available for the study species^29^.

For *K* = 3 and *K* = 2, 4 and 5, there is either zero or minimal admixture between *C.* *liberica* and *C.* *dewevrei*. Low-to-moderate admixture at *K* = 3 for the accessions from Ivory Coast, Ghana and Nigeria (Fig. 2b) suggest features of historical evolutionary processes and shared common ancestry (with *C.* *klainei*), rather than recent introgression. *C.* *liberica* and *C.* *klainei* are distinctly allopatric, with populations separated by ca. 800 km (Fig. 3). There is no evidence of any introductions of *C.* *klainei* to upper West Africa, which rules out the possibility of human-assisted introgression. One of the cultivated samples (23H79; Nigeria, Lagos, 1895, Millen 192) with the highest admixture (Fig. 2b) predates the introduction of *Coffea* germplasm into upper West Africa (Supplementary Text). Sample 23H79 and 23C17 (Ivory Coast, Abidjan, 1963, De Wilde 156) are both cultivated, but samples 23C16 (Nigeria, Omo, 1946, Jones & Onochie 17214) and 23C13 (Ghana, Atewa Range Forest Reserve. 1963, Enti & Jenik 36571) are of wild origin. For *K* = 4 and *K* = 5, these four accessions retain admixture (Supplementary Fig. 3) but not with *C.* *klainei*. The admixtures observed for *K* = 3 (Fig. 2b) probably account for the low QS (0.35) and LPP (0.50) scores in the *C.* *liberica* clade from the phylogenomic analysis (Fig. 1), despite the clade achieving a high BS value (100) (Supplementary Fig. 1).

## Morphological delimitation

**Extended Data Fig. 1:**
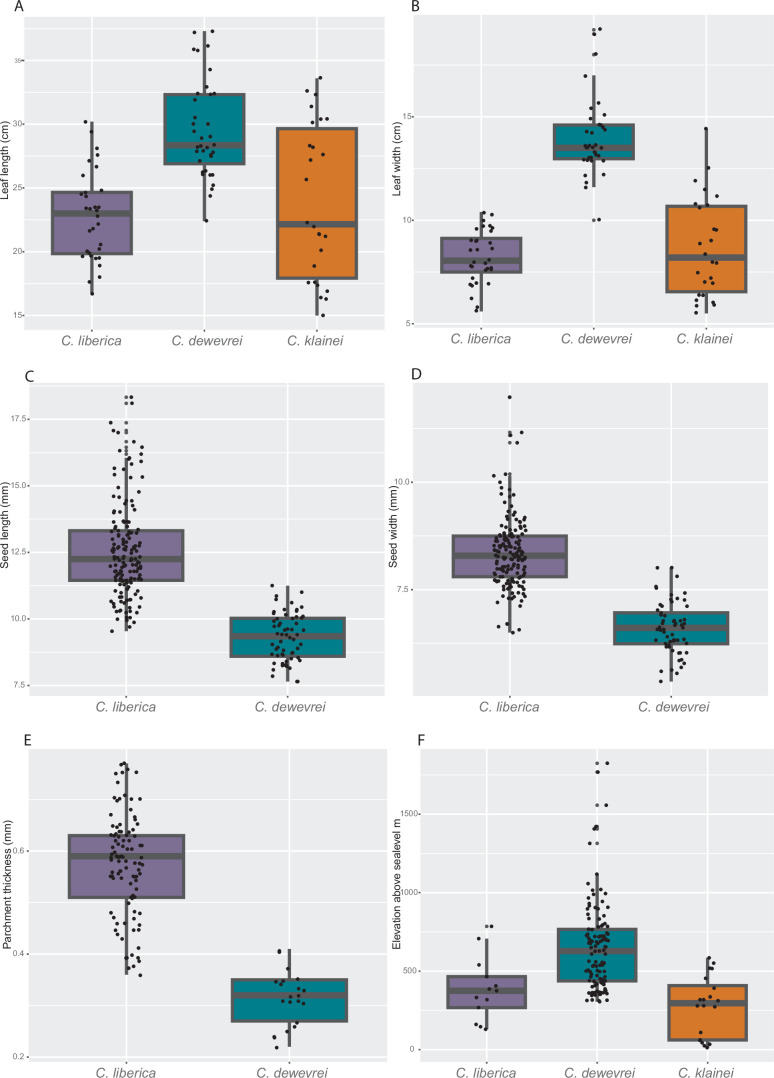
Box and whisker plots for length and width of leaves and seeds, parchment (endocarp) thickness, and elevation for *C. liberica* (Liberica), *C. dewevrei* (excelsa) and *C. klainei.* Mean values in square brackets; quartile values are given in Supplementary Information Table 2. **a**, **Leaf length (cm)**: *C. liberica* (22. 8 cm), *C. dewevrei* (29.5 cm) and *C. klainei* (23.9 cm). **b**, **Leaf width (cm)**: *C. liberica* (8.2 cm), *C. dewevrei* (14 cm) and *C. klainei* (8.7 cm). **c, Seed length (mm)**: *C. liberica* (12.6 mm) and *C. dewevrei* (9.3 mm). **d, seed width (mm)**: *C. liberica* (8.4 mm), *C. dewevrei* (6.6 mm). **e, Parchment thickness (mm)**: *C. liberica* (0.57 mm) and *C. dewevrei* (0.31 mm). **f, Elevation (m)**: *C. liberica* (386 m), *C. dewevrei* (654 m) and *C. klainei* (274 m). Overview of *t*-Tests: *C. liberica* and *C. dewevrei* are significantly different (*p* < 0.0005) for all six variables; *C. liberica* and *C. klainei* are not significantly different for any variables*; C. dewevrei* and *C. klainei* are significantly different for leaf length and width (*p* < 0.0005) and elevation (*p* < 0.005). *t-*Test results and other information are given in Supplementary Information Table 2.

**Extended Data Fig. 2:**
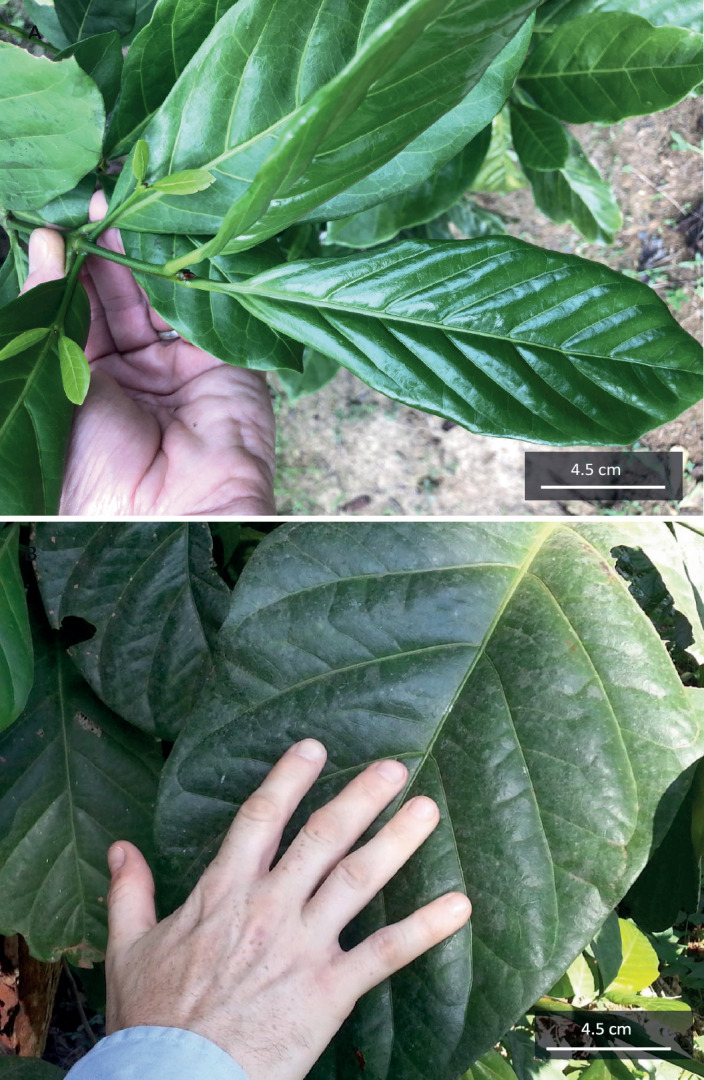
Leaves of *C. liberica* (Liberica) and *C. dewevrei* (excelsa). **Top**, *C. liberica*, shoot with leaves; **bottom**, *C. dewevrei*, leaves.

**Extended Data Fig. 3:**
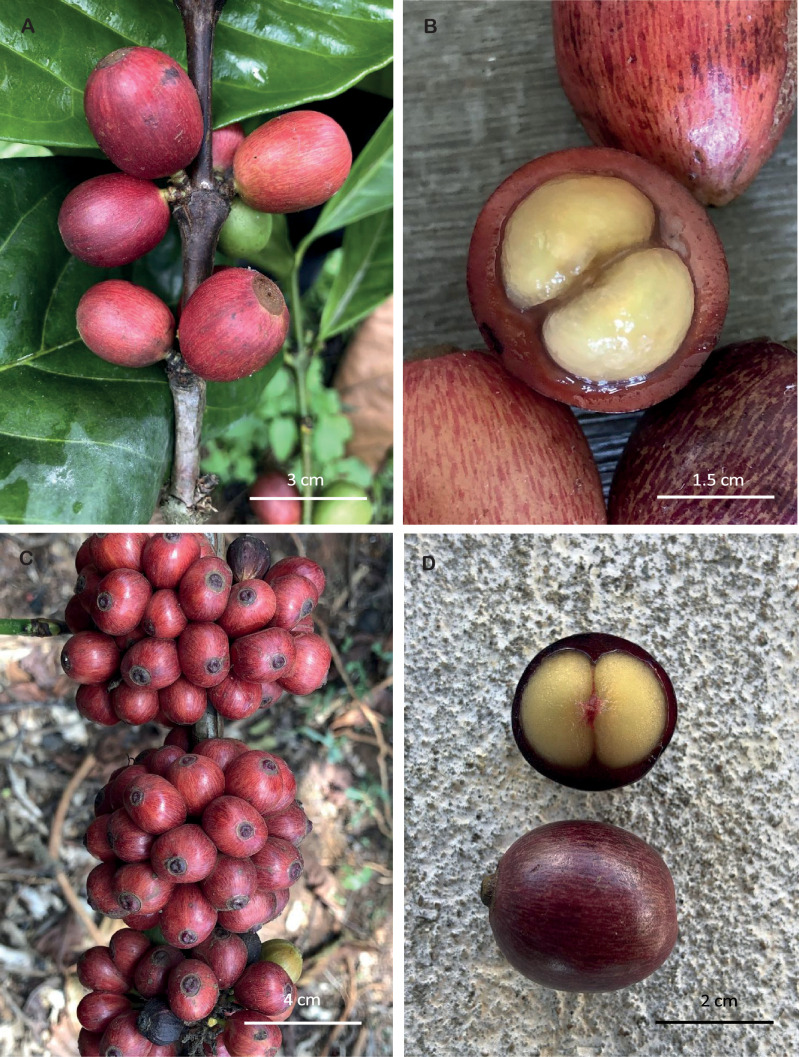
Fruits of *C. liberica* (Liberica) and *C. dewevrei* (excelsa). **a**, *C. liberica*, infructescences and fruits; **b**, *C. liberica*, fruit in partial cross section (pulp removed showing pyrene and outer surface of endocarp, with whole fruits. **c**, *C. dewevrei*, infructescences and fruits; **d**, *C. dewevrei*, fruit in partial cross section (above) with pulp removed and showing pyrene and outer surface of endocarp, and whole fruit (below).

**Extended Data Fig. 4:**
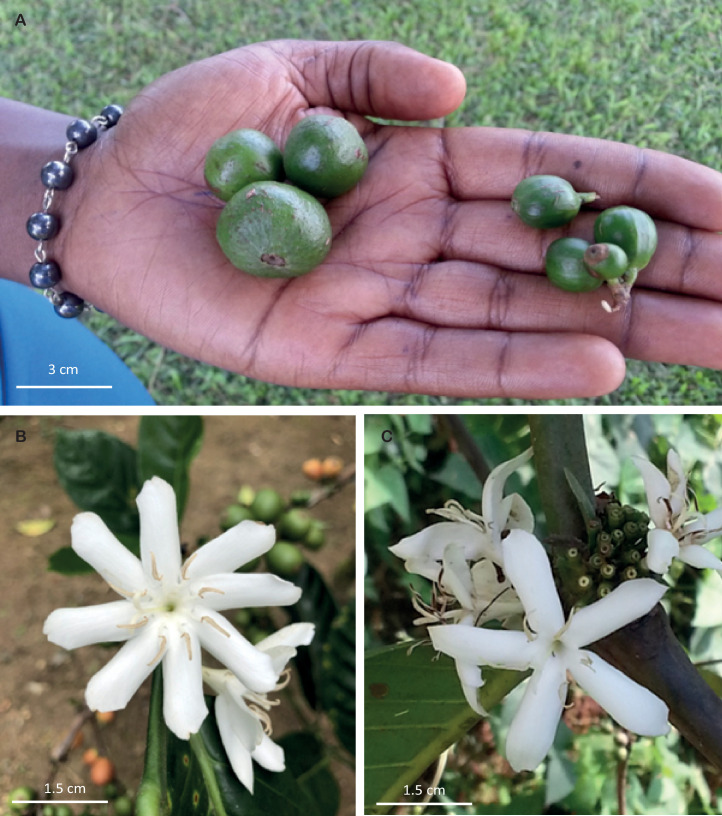
Fruits and flowers of *C. liberica* (Liberica) and *C. dewevrei* (excelsa). **a**, immature fruits: *C. liberica*, left and *C. dewevrei*, right; **b**, *C. liberica*, with 8-lobed (8-merous) flower; **c**, *C. dewevrei*, with 5-lobed (5-merous) flower. Flower size and shape is highly variable in both species.

We found that *C.* *liberica*, *C.* *dewevrei* and *C.* *klainei* are readily distinguishable using morphological characteristics (Table 1, Extended Data Figs. 1–4 and Supplementary Table 2). Compared with *C.* *liberica*, *C.* *dewevrei* has longer, broader leaves, more flowers (and thus more fruits) per leaf axil and node, fewer corolla lobes and flower parts per flower (usually five-merous flowers, versus six to nine-merous or more in *C.* *liberica*), smaller fruits with a thinner pulp (mesocarp), a thinner parchment (endocarp) and smaller seeds^2^ (Table 1, Extended Data Figs. 2–4 and Supplementary Table 2). The micromorphology of the seed epidermis and seed chemistry (diterpenes) may provide additional support for the separation of these two species^10^. *C.* *klainei* has a greater morphological affinity with *C.* *liberica*, but differs in having sessile, unbranched (as opposed to cymose and branched), inflorescences, with few flowers/fruits per inflorescence (usually one to three, versus two to six) and ellipsoid to narrowly ellipsoid fruits (versus spherical to ellipsoid). Experienced coffee professionals (for example, farmers and coffee buyers) can readily distinguish Liberica and excelsa based on the leaf dimensions (mainly overall size and shape and width), the fruit and seed size and yield (few or many per branch), in agreement with the morphological data presented here (Table 1 and Extended Data Figs. 1–4).

**Table 1:** Morphological characters distinguishing *C.* *liberica* (Liberica), *C.* *dewevrei* (excelsa) and *C.* *klainei*

Characters	*C. liberica* (Liberica)	*C. dewevrei* (excelsa)	*C. klainei*
Leaf size (length × width)* (mean values)	16.7–30.2 × 5.6–10.4 cm (22.8 × 8.2 cm)	22.4–35.9 × 10–18 cm (29.5 × 14 cm)	15–33.6 × 5.5–14.4 cm (23.9 × 8.7 cm)
Leaf shape	Elliptic to narrowly elliptic or obovate elliptic, rarely narrowly obovate elliptic	Elliptic to broadly elliptic or elliptic obovate	Narrowly oblanceolate to oblanceolate, rarely obovate to elliptic
Inflorescence type	Cymose (branched), very rarely single or fasciculate	Cymose (branched)	Single or fasciculate
Number of inflorescences per axil	(1–)2–4	(2–)3–6	1–2(–3)
Number of flowers per inflorescence	(1–)2–6	(2–)4–8	1–3
Number of flowers per axil	(2–)4–18(–24)	(4–)6–40(–48)	1–2(–3)
Number of corolla lobes (most frequent number)	6–9(–12) (7)	5(–6) (5)	7–8(–9) (7)
Fruit size (length × width)	(1.5–)1.8–3.4 × 1.7–3.3 cm	1.2–2.5 × 0.8–2.1 cm	2.8–3.5 × 1.4–2.2 cm
Fruit shape	Spherical to ellipsoid	Spherical to ellipsoid, rarely subglobose	Narrowly-ellipsoid to ellipsoid
Pulp (mesocarp) thickness	4–9.5 mm	2–3.5 mm	Not seen
Parchment (endocarp) thickness* (mean values)	0.36–0.77 mm (0.57 mm)	0.22–0.41 mm (0.31 mm)	ca. 0.6 mm
Seed size (length × width)* (mean values)	9.5–18.3 × 6.5–12 mm (12.6 × 8.4 mm)	7.7–11.3 × 5.4–8 mm (9.3 × 6.6 mm)	9–18.4 × 5.8–8.1 mm (12.2 × 7.2 mm)

*See Extended Data Fig. 1 for box and whisker plots for leaf width and length, seed width and length and parchment thickness. The number of flowers per inflorescence is usually greater than the number of fruits per infructescence, with a proportion of flowers not developing into mature fruits. One-flowered inflorescences are rare in *C.* *liberica* and usually the result of low light levels or restricted water and/or nutrient availability. Numbers in parentheses with an en-dash (–) indicate outlying or uncommon values.

## Indigenous distribution and elevation

After a thorough appraisal of ground point data (mostly for herbarium specimens) and focusing on the removal of cultivated and spontaneous (that is, self-sown into various habitats, but originating from cultivation) records, we demonstrate that the indigenous (wild) distributions of *C.* *liberica*, *C.* *klainei* and *C.* *dewevrei* are specific and allopatric (Fig. 3). *C.* *liberica* occurs in upper West Africa (Sierra Leone, Liberia, Ivory Coast, Ghana and Nigeria); *C.* *klainei* occurs in West-Central Africa (Cameroon, Gabon, the Republic of Congo and Angola (Cabinda)); and *C. dewevrei* in Central Africa (Republic of the Congo, Cameroon, the Democratic Republic of Congo, Central African Republic, South Sudan and Uganda). A Rapoport’s mean propinquity assessment^30^ using a barrier distance of 500 km demonstrates a robust population separation between *C*. *liberica* and *C.* *dewevrei* plus *C.* *klainei* (bold red line), but not between *C.* *dewevrei* and *C.* *klainei* (Fig. 3). The revised indigenous geographical range for *C.* *liberica* is comparable to that of two other *Coffea* species: *C.* *humilis* (Guinea, Sierra Leone, Liberia and Ivory Coast) and *C.* *stenophylla* (Guinea, Sierra Leone and Ivory Coast). This may infer shared drivers of *Coffea* species distributions in upper West Africa. *C.* *liberica* and *C.* *klainei* are predominantly located at low elevations (mean values of 386 m and 273 m, respectively), whereas *C.* *dewevrei* typically inhabits mid-elevations (mean of 653 m) (Supplementary Table 3).

## Climate parameters

Identifying the climate parameters essential for growth, yield and plant health is critical for optimizing the cultivation and development of crop species. *C.* *liberica* (Liberica) and *C.* *dewevrei* (excelsa) are recent introductions to agriculture (<200 years old)^2^, although wild gathering and local use may be date back centuries and perhaps millennia. At best, these two species are minimally domesticated; *C.* *klainei* is undomesticated and has only been cultivated in germplasm collections on a few occasions (Supplementary Text). Climate variable data for these species, over the natural distributions, provides a useful starting point for understanding climate requirements in cultivation^31,32^. Summary data for the 19 Bioclims^33^ are given in Supplementary Table 3.

The following narrative focuses on notable similarities and differences for the two crop species *C.* *liberica* (Liberica) and *C.* *dewevrei* (excelsa), as indicated in Supplementary Fig. 4 and Supplementary Table 4 (with *P* values). The annual mean temperature (Bio1) values are nearly identical (24.6 versus 24.4 °C), although Liberica has a lower mean diurnal range (Bio2; 7.9 versus 8.9). The main differences between *C.* *liberica* and *C.* *dewevrei* are for precipitation (the respective mean values are given): mean annual precipitation (Bio12; 2,215 versus 1,678 mm), precipitation of the wettest month (Bio13; 376.6 versus 230 mm); precipitation of the driest month (Bio14; 15.9 versus 36.2 mm); precipitation seasonality (Bio15; 66.9 versus 49.3); precipitation of the wettest quarter (Bio16; 988.9 versus 643.8 mm); precipitation of the driest quarter (Bio17; 80 versus 141.1 mm); and precipitation of the coldest quarter (Bio19; 961.7 versus 597.7 mm). Although the mean annual precipitation for *C.* *dewevrei* is lower than for *C.* *liberica*, *C.* *liberica* experiences lower precipitation in the dry season (Bio14 and Bio17). The higher mean annual precipitation for *C.* *liberica* is due to higher precipitation in the wet season (Bio16), which corresponds to the wetter coldest quarter (Bio 19). These patterns, along with precipitation seasonality (Bio15), are consistent with mean annual temperature and total annual precipitation climate bar charts. Upper West Africa (*C.* *liberica*) has a longer and more severe dry season with proportionally higher precipitation during the wetter/cooler months of the year, whereas much of the distribution range of *C.* *dewevrei* (in central Africa) has a shorter dry season, or dry seasons (if annual rainfall is bimodal), with a more even annual distribution of precipitation (Supplementary Tables 3 and 4). Comprehensive field trials for *C.* *liberica* and *C.* *dewevrei* are required to ascertain the precipitation requirements in cultivation and particularly to test whether *C.* *liberica* is better adapted to a more seasonal rainfall pattern and is perhaps more drought tolerant than *C.* *dewevrei*.

*C.* *dewevrei* has the greatest range for most of the Bioclims, which may infer either, or a combination of, the following: (1) that this species has greater climate plasticity, (2) that there is a wider range of climate tolerance over the entirety of the metapopulation or (3) simply that the considerably larger distribution (compared with *C.* *liberica* and *C.* *klainei*) encompasses a greater range of data values. *C.* *dewevrei* frequently occurs in riverine and gallery forest types within savanna woodland landscapes^2,3^, where populations may gain access to belowground or perhaps even surface water, at least during certain times of the year. Water availability in these habitats may enable this species to exist in areas of lower precipitation (for example, <1,000 mm yr^−1^), biasing precipitation values (for example, those for this species (that is, below Q1; Supplementary Fig. 4 and Supplementary Tables 3 and 4)). In the wild, *C.* *dewevrei* occurs in both open-canopy and closed-canopy forest^3^.

Under the revised circumscription and reassessment of wild occurrences, the mean annual temperature value for *C.* *liberica* is 0.7 °C higher (at 24.6 °C) than previously reported for *C.* *liberica* sensu lato (23.9 °C)^32^, and *C. dewevrei* is 0.4 °C higher (at 24.4 °C) than previously reported (23.9 °C)^32^. The modelled mean annual temperature for *C.* *liberica* is 5.9 °C and 0.9 °C higher than naturally occurring Arabica (*C.* *arabica*: 18.7 °C) and robusta (*C.* *canephora*: 23.7 °C)^32^, respectively; and for *C.* *dewevrei* (5.7 °C and 0.7 °C, respectively). The modelled mean total annual precipitation for *C.* *dewevrei* (1,678 mm) is similar to Arabica (1,614 mm) and robusta (1,699 mm)^32^, although field observations suggest that *C.* *dewevrei* exhibits a considerable measure of drought tolerance, particularly compared with *C*. *canephora*^2,3^. Ultimately, multilocation field trials over a range of climates, using a range of genotypes, would be required to more thoroughly compare climate tolerances for these four crop species.

## Implications for crop use and development

The species delimitations proposed in this study have implications for coffee crop development. Our genomic (Figs. 1 and 2) and phenotypic data (Table 1) reveal that *C.* *liberica* (Liberica) and *C.* *dewevrei* (excelsa) possess distinct alleles and unique allelic combinations of genes, as well as specific phenotypic (morphological) and climate characteristics. These attributes offer resources and utility for coffee breeding programmes.

The robust species delimitation identified enables the unambiguous partitioning of attribute data. For example, compared with *C.* *liberica*, *C.* *dewevrei* exhibits a higher yield^2^ owing to the number of fruits produced per tree, a higher outturn (that is, the conversion ratio of fresh fruit to clean (unroasted) coffee, mainly attributed to its thinner pulp^2^ and thinner parchment (endocarp)) (Table 1). In addition, the smaller seeds of *C.* *dewevrei* (Table 1 and Supplementary Table 2), which are similar in size and shape to Arabica^2^, make them more amenable to existing post-harvest, and preconsumption (roasting, packaging and coffee making) processes, as used for Arabica and robusta. Liberica and excelsa have contrasting coffee flavour profiles^2^, which influences consumer preferences and supports market differentiation. In addition to previously reported agronomic traits for these species, we report here that the parchment (endocarp) of *C.* *dewevrei* is conspicuously thinner than *C.* *liberica* (mean values of 0.31 versus 0.57 mm; Table 1, Extended Data Fig. 1 and Supplementary Table 2), which, in combination with a thinner pulp (mesocarp), improves outturns and ultimately the profitability of the harvested crop compared with Liberica.

Our climate analyses show that indigenous *C.* *dewevrei* is adapted to a lower mean annual rainfall, compared with *C.* *liberica* (Supplementary Fig. 4 and SupplementaryTables 3 and 4), but that *C.* *liberica* might be better adapted to higher precipitation seasonality and thus longer dry seasons, with periods (1–4 months) of relatively low precipitation. *C.* *liberica* occurs at lower elevations (mean of 386 m), whereas *C.* *dewevrei* is located at mid- to high elevations (mean of 386 versus 654 m; Supplementary Table 3). In cultivation, Liberica is mostly farmed at low elevations (10–500 m), in warm to hot (for example, a mean annual temperature of 24–27 °C) and wet (for example, mean annual precipitation of 2,000–4,000 mm) habitats, with low precipitation seasonality (short or indistinct wet season(s)), such as those in lowland regions of Malaysia, the Philippines and Indonesia. In contrast, excelsa is principally farmed at mid-elevations (500–1,200 m), with cooler temperatures (a mean annual temperature of 22–25 °C and with lower mean annual precipitation (1,500–1,800 mm), for example, in Guinea, South Sudan and Uganda. Notably, these species are rarely cultivated together or in overlapping agroecological zones. In tropical Central Africa^34,35^, Peninsula Malaysia and Sarawak (K. Lee Wing Ting, D. Jitam & A. Clayre, personal communication) and Java^36^ cultivated *C.* *liberica* flowers and fruits throughout the year. It is not certain whether this is the same for wild populations of *C.* *liberica*. By contrast *C.* *dewevrei* has a distinct flowering and fruiting seasons, although it is not known whether phenology would be disrupted under the conditions stated above for *C.* *liberica*.

Over their indigenous ranges, both species are likely to include populations with adaptations to regional climate differences, other abiotic factors (for example, soil), and various biotic interactions (for example, pest and disease incidence and resistance). This may particularly be the case for *C.* *dewevrei*, which has a large natural distribution range across tropical Central Africa. Importantly, Liberica and excelsa hold substantial potential for developing coffee farming in areas that are unsuitable for Arabica or robusta^2,32^, particularly those at low elevations in hotter and wetter climates (with higher mean annual temperatures and different annual precipitation patterns, see above). They may also have potential as a replacement coffee crop in areas that are becoming climatically unsuitable for Arabica and robusta. Excelsa has been used to replace robusta in some areas of Uganda, probably as result of climate change, for example, refs. ^2,3^.

Given the close phylogenomic relationship between *C.* *liberica* and *C.* *dewevrei* (Fig. 1) the production of fertile interspecies hybrids is likely^37^, either artificially (by hand or close-proximity cross pollination) or by chance^38^. *C.* *dewevrei* × *C.* *liberica* hybrids have been reported to be of outstanding vigour and yield^38^, although the existence of hybrids has not yet been verified by molecular methods. The use of either species in interspecies breeding programmes with other species may hold promise^3^.

Online sources regularly state that ‘Liberica’ (that is, *C.* *liberica* and *C.* *dewevrei*) provides 1–2% of the global coffee supply. This is incorrect, as these percentages are based on figures from the late-nineteenth century, when *C. liberica* stood with Arabica as the second most important coffee of commerce^2,39^. By the mid-twentieth century, Liberica was reported to represent about^40^ or less than^41^ 1% of global production, respectively. Today, global production of *C.* *liberica* and *C.* *dewevrei* is probably less than 1,000 metric tons (mt). Based on the figures for global exports of Arabica and robusta, which combined was around 10 million mt for 2024^1^, an estimate of production of 1,000 mt would represent 0.01% of global coffee exports. Despite this seemingly insignificant figure, Liberica and excelsa production is now being upscaled^2^, particularly in Uganda, South Sudan, India, Vietnam, Malaysia, the Philippines, Indonesia and even the Pacific.

## Extinction risk

Under our revised taxonomic circumscription, and refinement of indigenous distribution (Fig. 3), the extent of occurrence (EOO) for *C.* *liberica* decreases from 6,812,900 km^2^, as per the existing International Union for Conservation of Nature (IUCN) Red List assessment^42^, to 352,310 km^2^, which represents a reduction of 94.8%. The area of occurrence (AOO), is similarly affected, decreasing from 736 km^2^ to 52 km^2^, a reduction of 92.9%. The current IUCN Red List assessment reports that *C.* *liberica* occurs naturally in 17 countries^42^; our revised species delimitation reduces this to five: Sierra Leone, Liberia, Ivory Coast, Ghana and Nigeria. In all these countries, except Liberia, forest loss has been ongoing and in some cases severe, even over the past two decades^43^. It should be made clear, that the conservation metrics given above are based on historical records only, mainly from 1900–1980. Over the past 45 years, many of the populations and subpopulations recorded during that period have been extirpated or reduced in area and health owing to deforestation and other land-use changes. For example, in Sierra Leone, targeted searches for wild coffee species at Kasewe Hills Forest Reserve in 2023 and 2024 failed to locate *C.* *liberica* (Lebbie personal communication, 2024). To our knowledge, it was last recorded at Kasewe Hills in 1913 (herbarium specimen: Lane-Poole 128, 1913, K). Conversely, there are likely to be additional populations and subpopulations in Liberia, which has considerably more natural forest than the other four countries and is under-botanized. Given an AOO of less than 2,000 km^2^ (52 km^2^ for *C.* *liberica*), evidence of severe fragmentation (via Google Earth imagery) and continuing declines in AOO and EOO, *C.* *liberica* may warrant reassessment as a species threatened with extinction, shifting from its current classification of ‘Least Concern’^42^ to ‘vulnerable’ (VU B2 (a,b(i–v)))^44^. *C.* *klainei* has an AOO of 76 km^2^, and might also qualify as ‘Vulnerable’ under IUCN Red List criteria^44^, although further data would be required, for both species, before confident IUCN extinction threat assessments could be made. With an EOO of 2,464,990 km^2^ and an of AOO of 456 km^2^, *C.* *dewevrei* falls into the ‘Least Concern’ category, even with the observed forest loss evident in many areas of its distribution^3^. Regardless of the conservation metrics presented here, enhanced conservation measures are urgently needed for all three species, and particularly for *C.* *liberica*, to ensure their survival in the wild and potential role in global coffee sustainability.

## Methods

### DNA sampling

We sampled 12 accessions of *C.* *liberica* and 22 of *C.* *dewevrei*, sourced from wild and cultivated populations (farmed or germplasm collections), and 15 accessions representing all eight closely related species, as identified by prior molecular phylogenetic analyses^6,22,25^ and taxonomic study^5,16,24^. Material representing the geographical range of validly published synonyms for the two foci species were included in the DNA sampling (Supplementary Table 1 and Supplementary Text 1 and 2). Outgroup taxa from within *Coffea* (five accessions) were selected based on previous molecular phylogenetic analyses^6,12,22,25^. DNA was extracted from 26 herbarium leaf tissue samples, 13 silica-gel dried samples and 16 seed samples. Sampling details, other accession information and sequence information are provided in Supplementary Table 1. Accepted botanical names and authorities follow the International Plant Names Index (https://www.ipni.org).

### DNA sequencing and phylogenomic analysis

Total DNA was extracted using a modified CTAB protocol for herbarium specimens^45^. Sequence target capture data were generated using the universal Angiosperms353 target capture kit developed to retrieve 353 nuclear genes across the angiosperms^17,18^. Genomic libraries were constructed using an optimized protocol^46^ for half volumes of the NEBNext Ultra II DNA Library Prep kit for Illumina (New England Biolabs) and purified using AMPure XP magnetic beads and multiplexed using NEBNext Multiplex Oligos for Illumina (Dual Index Primer Sets I and II). Pools containing 55 genomic libraries mixed in equimolar conditions were enriched with half reactions of the Angiosperms353 probe kit following the myBaits kit manual v.3.02 (Arbor Biosciences), using an optimized protocol^47^. The DNA concentration and fragment size distribution were calculated using a Quantus fluorometer (Promega Corp.) and an Agilent 4200 TapeStation (Agilent Technologies), respectively. Sequencing was performed on a HiSeq (Illumina Inc.) by Macrogen, producing 2× 150 bp paired-end reads. Raw reads were submitted to the European Nucleotide Archive (https://www.ebi.ac.uk). ID codes are given in Supplementary Table 5. Trimmomatic v.0.35 (ref. ^48^) was used to discard low-quality reads and trim adaptors based on the reports generated by FastQC v.0.11.7 (ref. ^49^) and HybPiper v.2.3.0 (ref. ^50^) to retrieve the 353 nuclear loci using a combination of map to reference and de novo assembly methods for all samples. Alignments were generated in MAFFT 7.305b (ref. ^51^) using the command ‘auto’, then edited with trimAL v.1.4.rev22 (ref. ^52^) using the ‘automated1’ parameter.

Phylogenetic analyses were conducted for the concatenated and partitioned dataset of nuclear data (that is, the supermatrix approach) and by estimating a species tree from individual phylogenetic trees reconstructed for each nuclear locus independently (that is, multispecies-coalescent approach). Phylogenetic trees were reconstructed using RAxML-NG^53^ and IQ-TREE^54^ with 1,000 BS replicates. ModelFinder determined the optimal substitution model (-m MFP), selecting GTR + GAMMA^55^ on the best BIC score. Concatenated datasets were built with FASconCAT-G v.1.04 (ref. ^56^). ASTRAL‐III^57^ was used to construct a species tree based on the independent nuclear gene trees and the ASTRAL-III phylogenetic topology as input for the supermatrix approach. Support values were assessed using LPPs, with branches deemed supported if their LPP exceeded 0.95. To evaluate incongruence among gene trees, a quartet-based analysis was performed using the -t 8 option in ASTRAL-III, enabling the identification of the proportion of genes supporting alternative topologies at each node.

### SNP production and analyses

To generate SNP data for *C.* *liberica*, *C.* *dewevrei* and *C.* *klainei* (38 accessions), we used the framework developed by DePristo et al.^58^ using GATK^59^ following the pipelines established for Angiosperms353 data^21,60^. This process involved combining aligned and unaligned reads to a reference built with the longest exon obtained for all samples of the three species. We removed duplicate sequences and performed joint genotype calling for all samples after initially generating variants for each sample individually^61^ in a variant call format (VCF) file. The initial VCF file was processed with a stringent filter (QD < 5.0 || FS > 60.0 || MQ < 40.0 || MQRankSum < −12.5 || ReadPosRankSum < −8.0), removing indels and SNPs with missing data using GATK and eliminating linked SNPs with PLINK^62^. Base quality score recalibration was performed in GATK, followed by a repeated variant calling step.

To examine genetic differentiation patterns, we used STRUCTURE^26^ to determine the optimal number of genetic clusters in the dataset. STRUCTURE was run for five potential clusters (*K*), corresponding to the number of assumed genetic groups plus two. Each *K* was analysed with ten replicates, using 100,000 burn-in iterations followed by 1,000,000 Markov chain Monte Carlo repetitions. The most likely number of clusters was identified using Structure Harvester^27^, which implemented the method of Evanno et al.^28^ to calculate Δ*K* values. The *K* with the highest Δ*K* value was selected as one probable assessment of the number of clusters, and *K* = 3 was examined as an alternative, based on the number of clades obtained with the phylogenomic analysis (Fig. 1), genetic distance phylogenetic tree (Fig. 2a) and geographical distribution, heeding the issues raised regarding *K* = 2 and recommendations for exploring population subdivision^29^. *K* = 2, 4 and 5 were also examined. PLINK outputs were converted into STRUCTURE-compatible files using PGDSpider^63^. The results from STRUCTURE were visualized with StructRly^64^. Genetic differentiation was explored using a genetic distance-based phylogenetic tree using upgma with poppr 2.9.6, adegenet, ape 5.7.1 and RColorBrewer packages (Fig. 2a) and PCoA in R 4.3.0 (ref. ^65^), with the adegenet and ggplot2 packages, retaining three principal components to investigate genetic groupings (Fig. 2c).

### Morphological study

Morphological characters (Table 1) were measured from herbarium specimens (held at The Natural History Museum, London, UK (BM); Meise Botanic Garden, Belgium (BR); Royal Botanic Gardens, Kew, UK (K); Muséum National d’Histoire Naturelle, Paris, France (P) and Naturalis Biodiversity Center, Leiden, Netherlands (WAG)^66^) and living plants. More than 700 herbarium specimens, encompassing wild and cultivated *C.* *liberica*, *C.* *dewevrei* and *C.* *klainei*, were examined. Material representing all validly published synonyms of the three taxa were comprehensively studied (Supplementary Text 1 and 2). Living plants were studied in the wild and in cultivation settings across Africa, Madagascar and Asia. Parchment measurements were taken using a Mitutoyo no. 193-111, 0–25 mm (0.001 mm) micrometer. Seed measurement data were taken from published work^2,36^.

### Distribution and conservation assessment metrics

Data for the production of the distribution map, climate profiling (see ‘Climate profiling’ section) and conservation metrics for *C.* *liberica*, *C.* *dewevrei* and *C.* *klainei* were gathered from occurrence data points representing indigenous (wild) locations derived from herbarium specimens (BM, BR, K, MHU, P and WAG^66^) and field surveys. Georeferencing was performed for locations lacking coordinates, followed by manual validation and correction using Google Earth imagery. The dataset comprised 311 records, including 19 for *C.* *liberica*, 267 for *C.* *dewevrei* and 25 for *C.* *klainei*; after the removal of duplicate locations (within 1 km of each other), this was reduced to 152 data points (13, 119 and 20, respectively). The distribution map (Fig. 3) was produced in ArcGIS Pro 3.2.0 (Environmental Systems Research Institute) using Natural Earth (https://www.naturalearthdata.com) and their terrain and country boundaries dataset (version 5.1.1). IUCN Red List conservation metrics^44^, were produced using ShinyGeoCAT^67^, with default settings aligned to IUCN methodology and criteria^44^, providing the EOO (that is, a minimum convex polygon enclosing all occurrences) and AOO based on at least one occurrence in a 2 × 2 km grid cell (that is, the IUCN default^44^). A Rapoport’s mean propinquity assessment^30^ with a barrier distance of 500 km was used to test for population and subpopulation separation.

### Climate profiling

To understand the key climate parameters for each species, the statistics package R^65^ was used to sample the same dataset as above, against 19 Bioclim variables^33^ from the CHELSA dataset^68^. We reviewed all 19 Bioclim variables (Supplementary Fig. 4 and Supplementary Tables 3 and 4). For validation purposes, the modelled Bioclim data were compared against publicly available mean annual temperature and total annual precipitation climate bar charts.

### Reporting summary

Further information on research design is available in the Nature Portfolio Reporting Summary linked to this article.

## Supplementary information

Supplementary Information Supplementary Figs. 1–4, Tables 1–5 and Text 1 and 2.

Reporting Summary

Supplementary Tables 1–5 Supplementary Table 1. List of samples with key accession data and sequencing details. Supplementary Table 2. *t*-test results for morphological data. Supplementary Table 3. Summary of Bioclim data and elevation for *C. liberica*, *C. dewevrei* and *C. klainei*. Supplementary Table 4. *t*-test results for Bioclim data for *C. liberica*, *C. dewevrei* and *C. klainei*. Supplementary Table 5. European Nucleotide Archive ID and file codes.

## Data Availability

Raw reads for Angiosperms353 sequence data are available at the European Nucleotide Archive (https://www.ebi.ac.uk) under project no. PRJEB78707; ID codes are given in Supplementary Table 5.
