# Supplementary material for: Genomic data define species delimitation in Liberica coffee with implications for crop development and conservation
Source: Nat Plants. 2025 Aug 8;11(9):1729–38. doi: 10.1038/s41477-025-02073-y (PMC12449269; doi:10.1038/s41477-025-02073-y)
Supplement: Supplementary file 1 — Supplementary Figs. 1–4, Tables 1–5 and Text 1 and 2. [file 41477_2025_2073_MOESM1_ESM.pdf]

nature plants

Supplementary information https://doi.org/10.1038/s41477-025-02073-y

Genomic data define species delimitation in Liberica coffee with implications for crop development and conservation

In the format provided by the authors and unedited

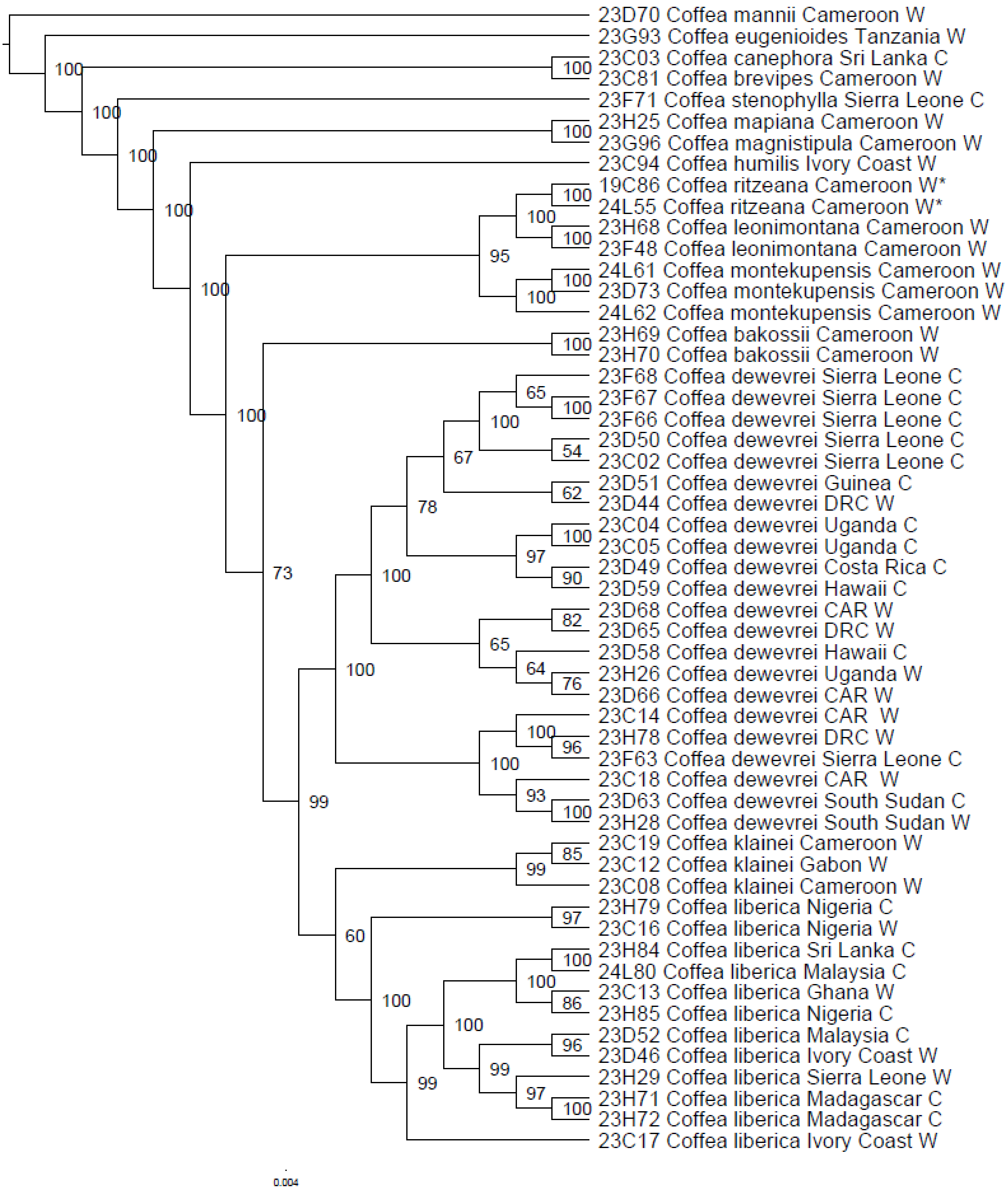

Supplementary Fig. 1. Supermatrix tree with bootstrap support values (including *C. magnistipula*). Codes preceding species names represent DNA library code. C=cultivated; W=wild (indigenous). See Supplementary Table 1 for accession details.

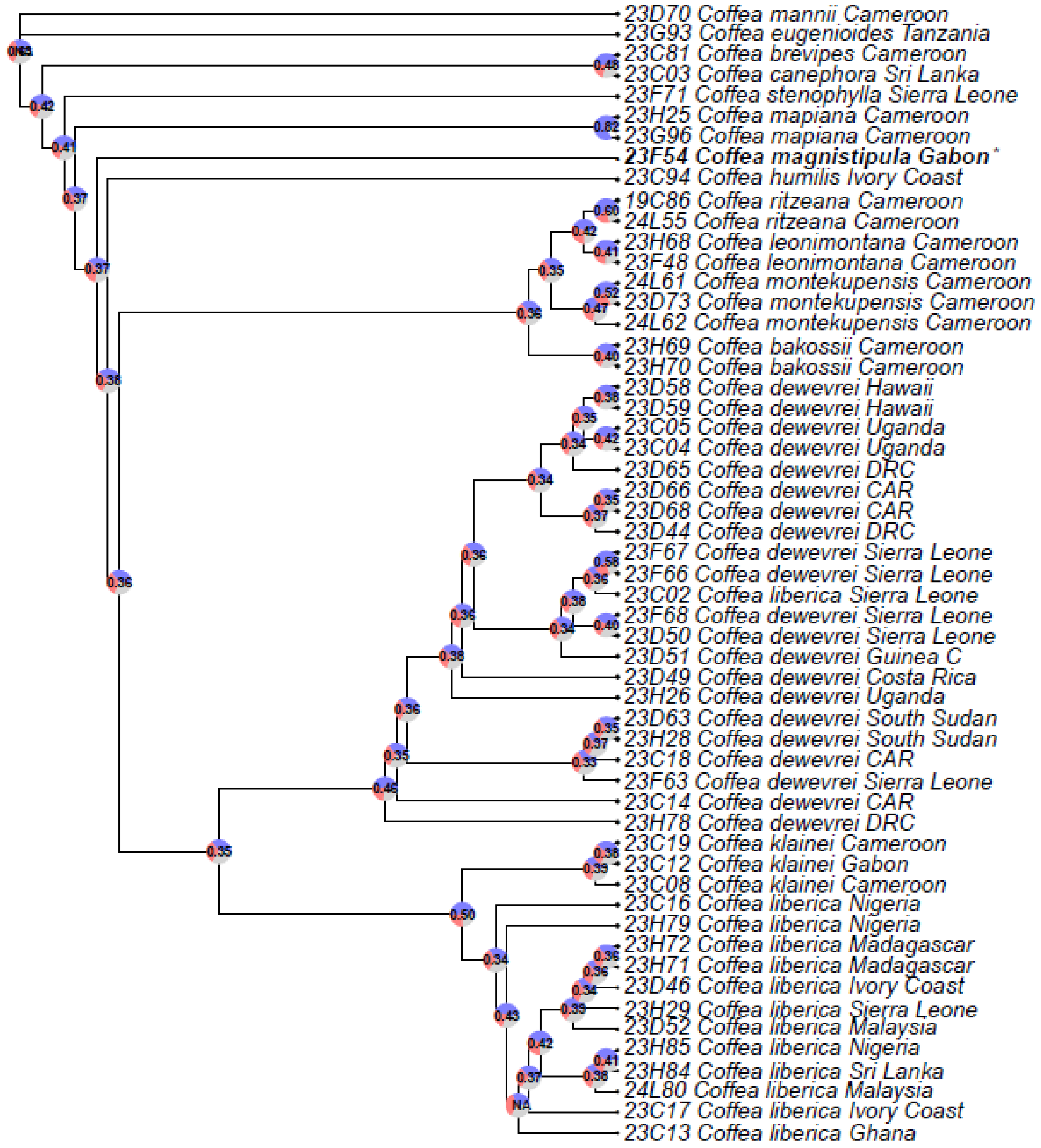

Supplementary Fig. 2. Astral tree with inclusion of *C. magnistipula*. Pie charts show quartet scores (QS) informing on the agreement between genes. *Coffea magnistipula* indicated in bold*.

K2

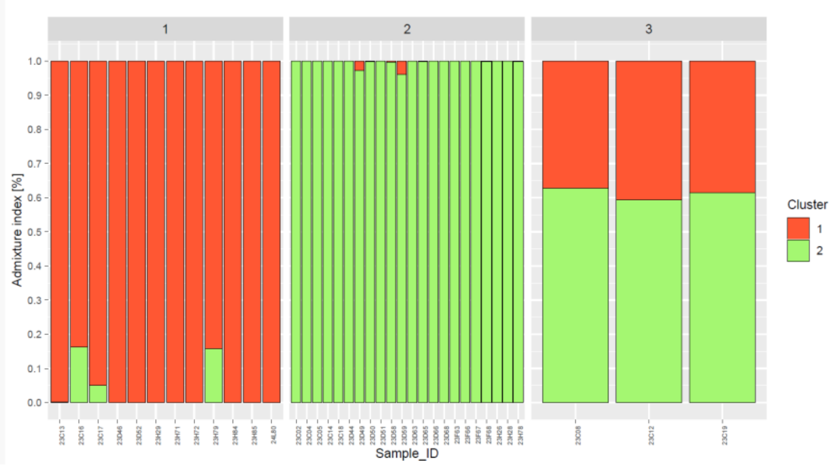

K4

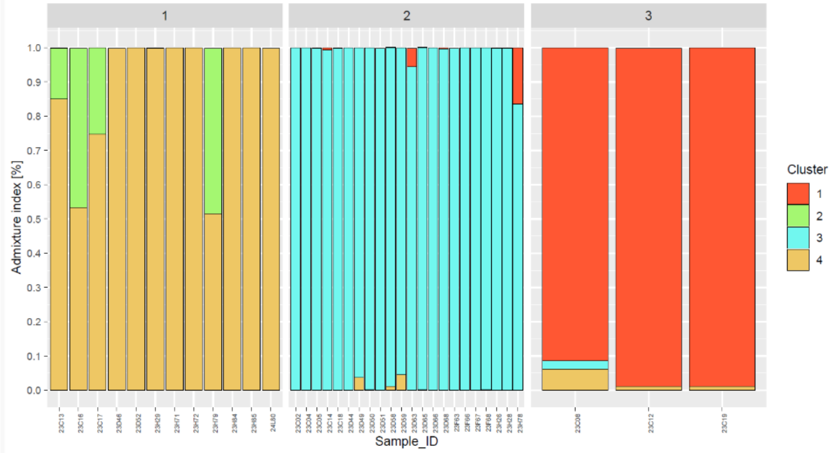

K5

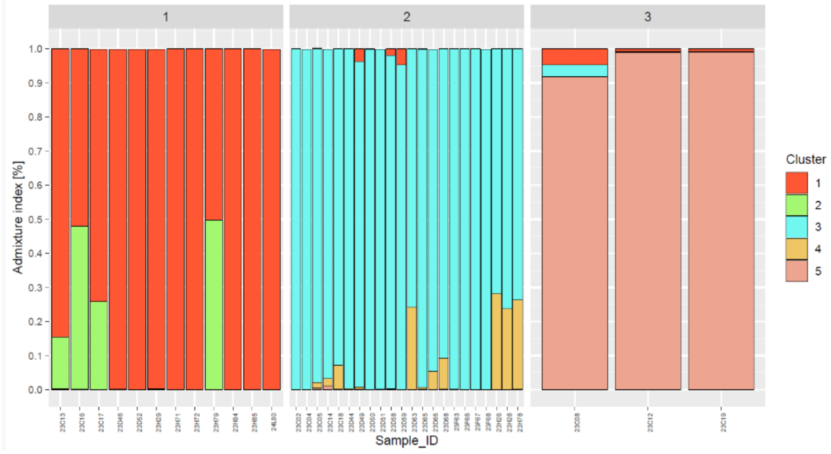

Supplementary Fig. 3. Alternative STRUCTURE[1] *K* values for *C. liberica*, *C. klainei* and *C. dewevrei*. Analyses based on 2,240 exon region SNPs for 37 samples. Group 1 = *C. liberica*; group 2 = *C. dewevrei*; group 3 = *C. klainei*. *K* values of *K*=2, 4 and 5. *K*=2 (ΔK value of 3605.7 compared to < ΔK 37.54 for the remaining *K* values assessed) represents the likely number of *K* genetic[2,3].

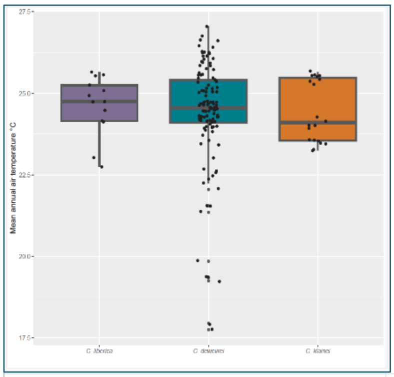

BIO1 = Annual Mean Air Temperature

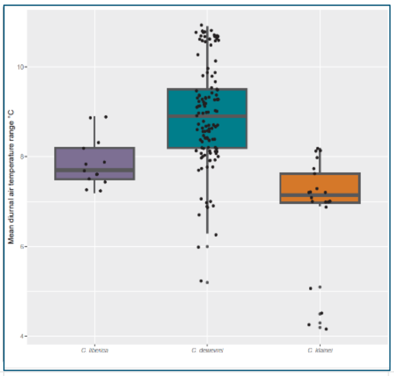

BIO2 = Mean Diurnal Air Tempterture

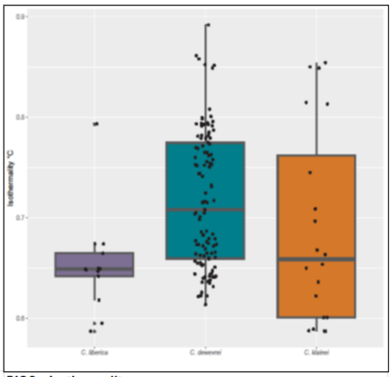

BIO3 = Isothermality

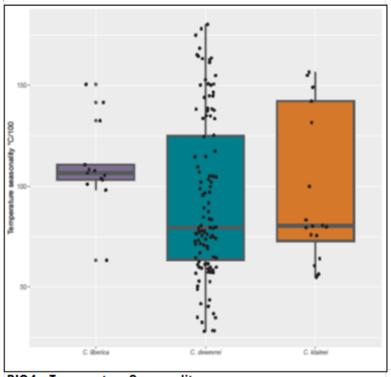

BIO4 = Temperature Seasonality

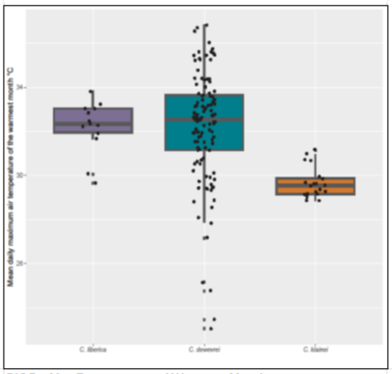

BIO5 = Max Temperature of Warmest Month

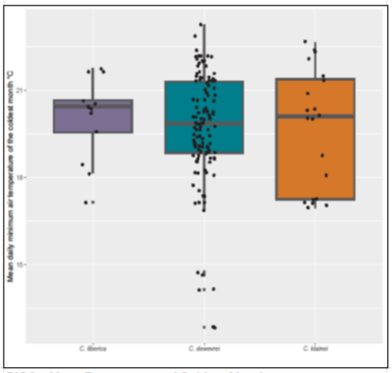

BIO6 = Mean Temperature of Coldest Month

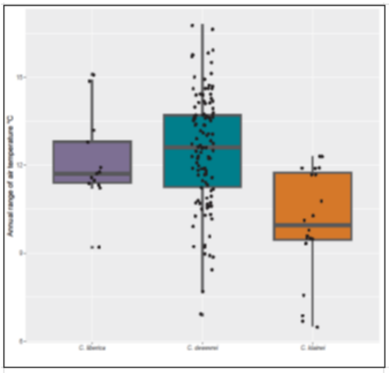

BIO7 = Temperature Annual Range (BIO5-BIO6)

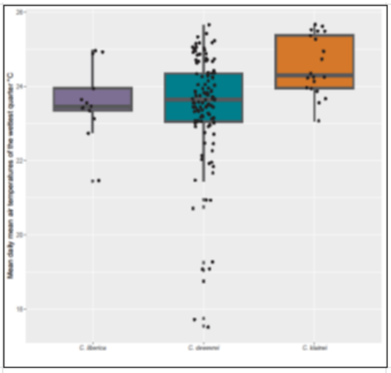

BIO8 = Mean Temperature of Wettest Quarter

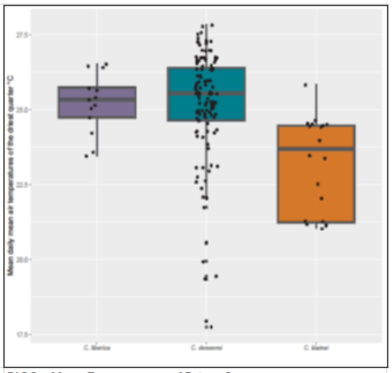

BIO9 = Mean Temperature of Driest Quarter

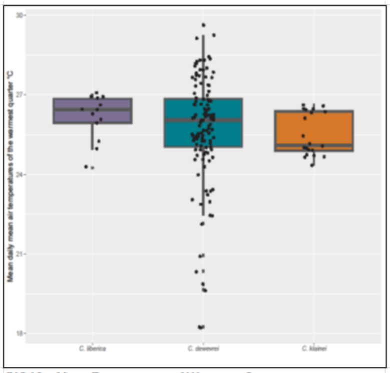

BIO10 = Mean Temperature of Warmest Quarter

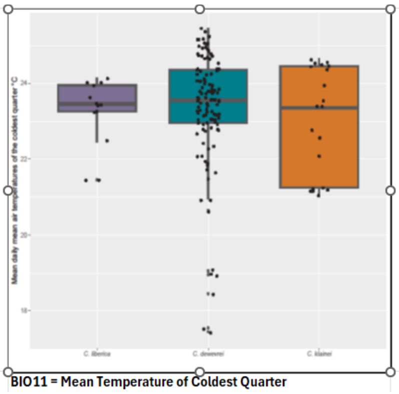

BIO11 = Mean Temperature of Coldest Quarter

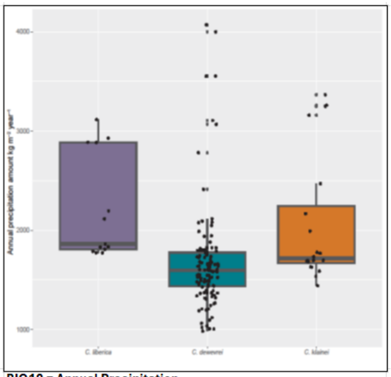

BIO12 = Annual Precipitation

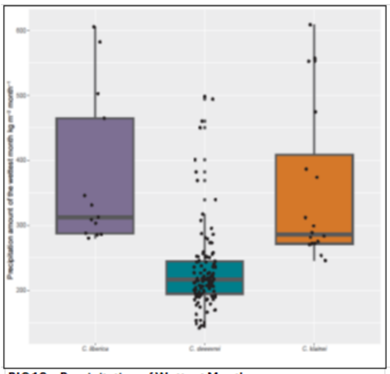
BIO13 = Precipitation of Wettest Month

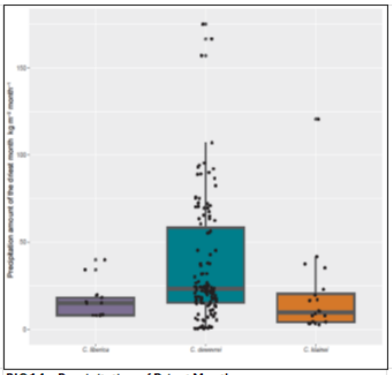
BIO14 = Precipitation of Driest Month

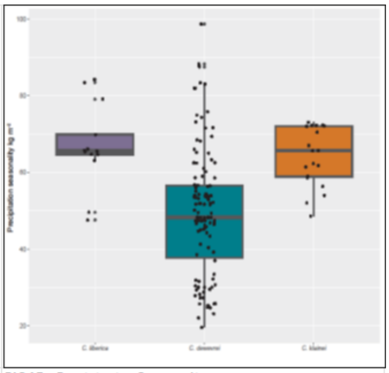
BIO15 = Precipitation Seasonality

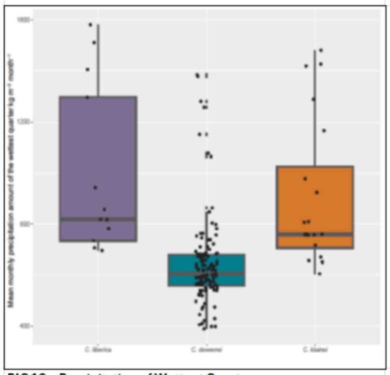
BIO16 = Precipitation of Wettest Quarter

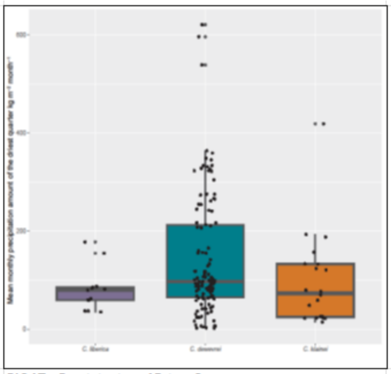
BIO17 = Precipitation of Driest Quarter

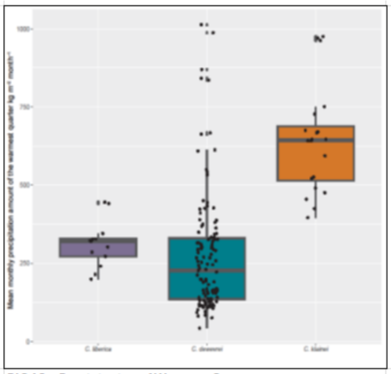
BIO18 = Precipitation of Warmest Quarter

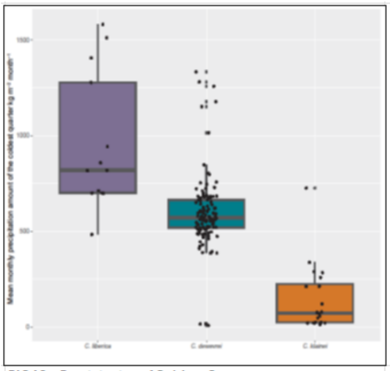
BIO19 = Precipitation of Coldest Quarter

Supplementary Fig. 4 Box and whisker plots for 19 Bioclim variables for *C. liberica*, *C. dewevrei* and *C. klainei*. See Supplementary Table 3 for data summary and Supplementary Table 4 for *t*-Test results.

Supplementary Text 1

Taxonomic history for *C. liberica*, *C. dewevrei*, and *C. klainei* and associated species

Coffea liberica

The first known mention of Liberica coffee (*Coffea liberica* W.Bull.) was in the Appendix to the Report of the Sierra-Leone Company, published in 1794; on page 173 Adam Afzelius (1750–1837) states: 'Coffee trees of two distinct species, both nondescript; but whether of any use is not yet ascertained'[4]. According to Hiern[4], the two species mentioned are *C. liberica* and *C. stenophylla* G.Don. *Coffea liberica* was formally described in 1874[5], based on plants from Liberia and Sierra Leone[6] in Upper West Africa. It was lectotypified in 1985[7], based on a specimen cultivated in Sierra Leone in 1836 (see Taxonomic treatment, below).

Well before the formal recognition of *C. liberica*, this species was traded during the early 1800s[8,9]. From the 1870s to 1900s it was widely disseminated across the tropical belt, as a replacement crop for Arabica coffee (*C. arabica* L.) during the coffee leaf rust (*Hemileia vastatrix* Berk. & Broome (1869) epidemic of the late nineteenth century[8,10,11]. *Coffea liberica* caught the attention of botanists and coffee growers due to its size, being a tree capable of reaching 10 m or more, and its large fruits and seeds[9].

Coffea dewevrei

In 1899 *C. dewevrei* De Wild. & T.Durand was described as a new species from the Democratic Republic of the Congo (DRC)[12]. Its affinities were said to be with *C. canephora*, due to its 5-lobed corolla (5-merous flowers) rather than *C. liberica*, *C. macrochlamys* K.Schum. and *C. hypoglauca* Welw. ex Hiern, which were reported as being 6–7-merous[12]. Of these three species, only *C. liberica* is now considered to be a *Coffea*, the other two species belong in different genera of the Rubiaceae: *Calycosiphonia macrochlamys* (K.Schum.)Robbr. and *Belonophora coffeoides* subsp. *hypoglauca* (Welw. ex Hiern) S.E.Dawson & Cheek, respectively (https://www.ipni.org). Following the description of *C. dewevrei*, there came the description of a plethora of related species, including *C. arnoldiana* De Wild. from the DRC; and *C. dybowskii* Pierre ex De Wild., *C. sylvatica* A.Chev., *C. excelsa* A.Chev., *C. aruwimiensis* De Wild., and *C. royauxii* De Wild., from the Central African Republic (CAR), as outlined below (Taxonomic treatment). Several other related species and botanical varieties were recognized but never validly published[13,14]. Chevalier later concluded that all the above species represented variability within *C. dewevrei*¸ and were reduced to varieties[15,16] and then races[17] of *C. dewevrei*.

Coffea klainei

Coffea klainei Pierre ex De Wild. was described as a new species in 1901, based on material collected in the forest of Sibang, near Libreville[18]. It was considered 'close to' *C. liberica*, differing by its large, ovate and cuspidate (i.e. leaf apex with a distinct point) leaves, sessile and large (3.7 cm long), 7- often 9-merous flowers, with a concave (nectary) disk, and suboblong, cuneiform (wedge-shaped) seeds[18].

The species was consistently recognized by Chevalier[15,17,19], although he did suggest that it might be a 'une remarquable mutation de *C. liberica* retournée à l'état sauvage [a remarkable mutation of *C. liberica* returned to the wild][19]. He also observed that the fruits, though usually distinctly ovoid may also be subspherical, and the seeds may be rounded at both ends rather than being distinctly pointed. Chevalier tasted the coffee of *C. klainei* and found it to be excellent, but stated that because of its large seeds it would not be very popular in France[19]. Review of herbarium specimens from Gabon, as captured in the illustrations of this species (see Taxonomic treatment) shows that *C. klainei* usually has large narrowly obovate or obovate leaves, although the leaf morphology is highly variable; the flowers few are few (1–2, sometimes 3) per inflorescence), usually large and sessile, and the fruits are sessile, large, ellipsoid to narrowly ellipsoid, borne in single or in few fruited (2, rarely 3) fascicles.

Coffea abeokutae

Coffea abeokutae Cramer[20], was described as a new species in 1913, based on material originally collected in the district of Abeokuta in Nigeria, then cultivated at the Royal Botanic Gardens, Kew (England), and sent to Java in 1899[20]. It was cultivated in the Cultuurtuin te Buitenzorg (now Bogor Botanic Garden), where it proved to be high yielding and hardy[21]. This new species had clear affinities with *C. liberica*, due to the shape and size of the leaves, and having flowers with 6–8 lobes (6–8-merous flowers). It differed due to its smaller flowers, smaller fruits ((1.3–)1.5–1.8(1.9–2) cm long[20]), softer fruit pulp (mesocarp), and smaller seeds (10.2–13.5 × 8.2–9.7 mm)[20]. *Coffea abeokutae* was included in the 1929 work by Chevalier but with little detailed information [19]. In 1937 Portères[22] mentions a first report of *C. abeokutae* in Ivory Coast in 1907, encountered by Chevalier near the village of Guidéko in the Middle Basin of the Sassandra River. It is not clear whether this record represented a wild, spontaneous or cultivated plant. In 1909 Chevalier found 'another form' of *C. abeokutae* in western Ivory Coast in the vicinity of Assikasso[22]. Thereafter it was frequently reported in several places in Ivory Coast, both spontaneous and cultivated, but with little reference to truly wild populations, other than those associated with *C. liberica* from the forests bordering Liberia[22-24]. In an attempt to provide formal recognition of the diversity (mainly for fruit size and shape) found in *C. abeokutae* in Ivory Coast, Portères established two varieties[22]: *C. abeokutae* var. *sphaerocarpa*, and *C. abeokutae* var. *longicarpa*. Later, Chevalier[17] added *C. abeokutae* var. *indeniensis*, and *C. abeokutae* var. *microcarpa, C. abeokutae* var. *camerunensis*[15,17] and *C. abeokutae* var. *macrocarpa*[17] from Cameroon, but none of these names were validly published. Most of these names were based on cultivated or naturalized plants. On review of the works of Portères[22-24] it is becomes apparent that much of the material seen and worked on by him was a mixture of potentially wild, naturalized, and cultivated plants related to *C. liberica* and identified as *C. abeokutae*, and also hybrids, probably between these plants and early (c. 1900 onwards) introductions of *C. dewevrei* (but under various names) from the Kisantu research stations in the then (now DRC) Belgian Congo[22]. The general names used by Portères for these plants in Ivory Coast was 'excelsoides' or 'Gros Indénié', as a means of differentiating coffees with large seeds from those of *C. canephora* Pierre ex A.Froehner, which have smaller seeds and were referred to as 'Petit Indénié' or 'Indénié à petits grains'. Seed size variability, and high percentages of pea berries ['caracoli'] (fruits containing a single rounded seed, formed in response to poor seed fertility) among many samples of

‘excelsoides’ or ‘Gros Indénié’ e.g. 23–50%, [22], infers that these were hybrid coffee plants; this was implied but not specifically stated by Portères[22,24] . For fully fertile coffee plants the percentage of pea berries is usually 10% or less, and for hybrid plants 25–100%[25].

Taxonomic opinions

In 1941, Lebrun[26] summed up what he considered to be the taxonomic consensus[16,19,27] for *C. dewevrei* and *C. liberica*, believing these species to represent two ‘races’, characterized by some morphological and geographical differences, but with insufficient diagnostic characters to distinguish them as species due to the numerous exceptions and intermediates. Two varieties were formally recognised and delimited: (1) *C. liberica* W.Bull. var. *liberica*: corolla 6–9-merous, with a very widened (flared) tube at the throat, with broad lobes, usually 5–10 mm wide; floral disk often short and thick, obconical, flared and rounded, sometimes ciliolate or puberulous at the top; fruit generally large, 20–25 × 17–21 mm. (2) *C. liberica* var. *dewevrei* (De Wild. & T.Durand) Lebrun: corolla 5–6-merous, exceptionally up to 7–8-merous, with a tube moderately flared at the throat, with narrower lobes, usually 2.5–7 mm wide; floral disk often cylindrical or truncated, generally denticulate or lobed at the top, rarely entire or puberulous towards the apex; fruit of moderate size, reaching 12–20 × 8–16 mm. Although Lebrun did not explicitly place previously described species under each of the two botanical varieties, it can be deduced from his list of specimens examined (*exsiccata*) that *C. aruwimiensis, C. arnoldiana*, *C. dybowskii*, *C. dewevrei*, *C. excelsa* and *C. royauxii* were treated by him as synonyms of *C. liberica* var. *dewevrei*; and *C. abeokutae, C. klainei* and *C. zenkeri* K.Krause ex De Wild. as synonyms of *C. liberica* var. *liberica*.

Chevalier[15,17] had a less reductionist view compared to Lebrun, as he maintained *C. liberica* and *C. dewevrei*, and recognized three closely related species, viz. C. *klainei*, the little known *C. oyemensis* A.Chev.[28], and *C. abeokutae*[15,17]. *Coffea klainei*[18] and *C. oyemensis*[28], two species described from Gabon, the former collected from the wild (see above) and the latter from a single cultivated individual said to have been originally collected from the wild in Gabon.

The current consensus of taxonomic and systematic study[13,26,29-35] is for *C. liberica* to be divided into two botanical varieties: var. *liberica* and var. *dewevrei*, as established by Lebrun[26], incorporating the associated species as listed above. An alternative viewpoint is that *C. liberica* is a single highly variable species without infraspecific taxa, encompassing most or some of taxa mentioned above as synonyms[36,37].

Review of the literature tells us that *C. liberica* has been widely distributed across the world, particularly in the late nineteenth and early twentieth centures[9]. Less is known about the dissemination history of *C. dewevrei* (under various names) and related species. Of particular importance for understanding species boundaries in *C. liberica sensu lato* are those accounts of introductions into or between the indigenous ranges of *C. liberica* and *C. dewevrei*. De Wildeman[38] stated that plants of *C. liberica* were introduced into the Congo (DRC) in 1888. Chevalier[39] reported introductions of *C. dewevrei* (under the name *C. excelsa*) from Chari (Central African Republic) to Guinea in Upper West Africa. In 1905 it was reported that naturally occurring (wild) *C. liberica* had not been encountered in Guinea, but that it would be likely to occur in areas on the border with Liberia[40]. *Coffea*

dewevrei (under the names *C. arnoldiana*, *C. dybowskii* and *C. excelsa*) was introduced into Ivory Coast during the early 1900s[22], and then with increasing frequency through the 1920s and 1930s[22,24]. Many of these introductions were reported as originating from the Kisantu research station in western DRC, and then disseminated via the agricultural research station at Bingerville (now a suburb of Abidjan)[22,24] in Ivory Coast. *Coffea dewevrei* (then as *C. excelsa*) was also an early introduction to several locations in Vietnam, in 1904 and 1905[40].

Based on the evidence provided in the main text, we have proposed the formal resurrection of *C. liberica* var. *dewevrei* to the rank of species, i.e. *C. dewevrei*, following the rules of nomenclatural priority[41]. Excelsa is the appropriate vernacular name for *C. dewevrei*, as it is in common and widespread use[9,35,42]. The common name Liberica or Liberian coffee should be used only for *C. liberica*. A great deal of the problems encountered in the past were no doubt due to lack of access to authentic material of known provenance. For example, some morphological comparisons of var. *liberica* and var. *dewevrei*, seem to have been based on *C. liberica* alone[31]. Molecular investigations have almost exclusively relied on cultivated material, which was either collected from the wild during the original introductions in the nineteenth century[9] or several decades ago (1960–1980). The corruption and loss of biological integrity for whole-plant coffee germplasm collections, through inadequate management (including accession record errors) and loss of biological integrity via outcrossing, is well documented[43-45]. The advantage of the Angiosperms353 target capture kit is that museum collections, and particularly herbarium collections of guaranteed provenance, can be used to generate considerable sequence coverage and capturing of genes for resolving phylogenomic relationships (see main text). Introduced plants of *C. liberica sensu lato* (i.e. var. *liberica* and var. *dewevrei* (or under other names; see above) have served to further confuse species boundaries in Upper and Central West Africa (particularly in Ivory Coast; see above), and in Asia.

Coffea klainei is a poorly know species, previously considered to represent a synonym of *C. liberica*[13,14]. It is closely related to *C. liberica* (see main text) but can be easily separated on the basis of morphological and genomic data, and its distribution range is separated from *C. liberica* by c. 800 km (see Fig. 3, main text). We formally propose that *C. klainei* be resurrected from synonymy as a distinct species.

With *C. dewevrei* and *C. klainei* reinstated, the total number of coffee species now stands at 133; Cameroon becomes the country with the highest number of indigenous *Coffea* species (18 species) in Africa (Tanzania: 17 species), second only to Madagascar (67 species) globally. (https://checklistbuilder.science.kew.org/reportbuilder.do).

Supplementary Text 2

Taxonomic treatment for *C. liberica*, *C. dewevrei* and *C. klainei*

1. *Coffea liberica* W.Bull., Retail List Beaut. & Rare Pl. 97: 4 (1874). Type: Cultivated at Sierra Leone, specimen obtained from Mr Effenhausen's farm, 8 i 1836, Daniell s.n. (lectotype BM!).

Synonym:
Coffea abeokutae Cramer, Meded. Dept. Landb. Ned. Indië 11(15): 286, 396 (1913). Type: Experimental Garden, Buitenzorg, Java, collector unknown, commissioned by *P.J.S Cramer* (holotype K!; 2 sheets). Note. The letter accompanying the type specimen, and the diagnoses, clearly state that this plant was originally derived from Abeokuta, Nigeria, and not from Ivory Coast, as stated by Chevalier (1920).

Illustrations: Hiern (1876, pl. 24)[4]; Cruwell (1878, pl. 1 & 2)[11]; De Wildeman (1907, pl 104)[46]; Cheney (1925, pl. 31)[47]; Chevalier 1929, fig. 11)[19]; Lebrun (1941: pl. 17, fig. 7)[26]; Chevalier (1942: pl. 1)[15] [the plate legend states 'Forme cultivé in Côte d'Ivoire', but the specimen cited (no. 23350 Herb. Chev.) represents a cultivated plant from Porto Novo, Benin]; Keay (1963: fig. 231)[36].

Literature: De Wildeman (1906: 338)[38]; Cheney (1925: 76–90)[47]; Chevalier (1947: 170)[17]; Bridson (1985: 806)[7].

Distribution: Sierra Leone, Liberia, Ivory Coast, Ghana, Nigeria. Fig. 3.

Ecology: Humid, evergreen forest; 130–500(—785) m.

2. Coffea klainei Pierre ex De Wild., Caféiers: 13 (1901). Type: Près de Libreville dans les forêts, xii 1900, *R.P. Klaine* 1838 (syntypes BR!, K!, MO!, P!) [According to De Wildeman (1901) "Fructifies in August, flowers in April (Pierre in Sched.)"].

Synonyms:
Coffea liberica var. *gossweileri* A.Chev., Rev. Bot. Appl. Agric. Trop. 19: 398 (1939). Type: [Angola (Cabinda)] Lufo Hombe-Maiombe, 2 iv 1919, *J. Gossweiler* 7972 (syntypes: COI, K!, BM!, LISC, MO).
Coffea oyemensis A.Chev., Rev. Bot. Appl. Agric. Trop. 19: 403 (1939). Type: ['Gabon: cultivé au poste d'Oyem, de grains provenant de l'forêt environnante circonsription du Wolen-Ntam, en fleurs, 5 i 1933'] Plantes recueillies dans la circonscription de Woleu-Ntem, *G. Le Testu* s.n. [b.876] (syntypes: BR!, BM!, P!). Note. In the protologue Chevalier (1939) states that the flowers as 5-merous (as per *C. dewevrei*), but the syntype at BR has 5-, 6- and 7-merous flowers. Chevalier (1939) states affinities with *C. klainei* and *C. abeokutae* (*C. liberica*). No fruiting specimens exist.

Illustrations: De Wildeman (1907, pl 102)[46]; Chevalier (1942, pl. 11, & pl. 41 [as *Coffea liberica* var. *gossweileri*])[15]

Literature: Chevalier (1929: 78)[19]; Chevalier (1947: 174)[17];

Distribution: Cameroon, Gabon, Republic of the Congo, Angola (Cabinda), Fig. 3.

Ecology: Humid, evergreen forest; (10–)60—440(—585) m.

3. **Coffea dewevrei** De Wild. & T.Durand, Bull. Soc. Roy. Bot. Belgique 38: 202 (1899). *Coffea liberica* var. *dewevrei* (De Wild. & T.Durand) Lebrun, Mém. Inst. Roy. Colon. Belge, Sect. Sci. Nat. Méd. (8vo) 11(3): 168 (1941). Type: [Democratic Republic of the Congo], Bema Lecoula, December 1896, *A.A. Dewèvre* 1149 (holotype BR!).

Synonyms:

Coffea arnoldiana De Wild., Compt. Rend. Congr. Intern. Bot. 1900: 236 (1900). Type: ['Cafèier de l'Aru, cultivé à Eala'] Bas Congo [Democratic Republic of the Congo], *A.A. Dewèvre* 377 (holotype BR!).

Coffea dybowskii Pierre ex De Wild., Caféiers: 14 (1901). *Coffea dewevrei* var. *dybowskii* (Pierre ex De Wild.) A.Chev., Encycl. Biol. 22: 29 (1942). Type: Congo Français [Central African Republic], forêts des bords du Kemo, 15 ii 1892, *J. Dybowski* 672 (holotype P!; isotypes K!, P!).

Coffea sylvatica A.Chev., Rev. Cultures Colon. 12: 258 (1903). *Coffea dewevrei* var. *sylvatica* (A.Chev.) A. Chev., Encycl. Biol. 22: 29 (1942). Type: Oubangui [Central African Republic], Bangui, 16 viii 1902, A. Chevalier 5200 (syntypes K!, P! (mult.).

Coffea excelsa A.Chev., Rev. Cultures Colon. 12: 258 (1903). *Coffea dewevrei* var. *excelsa* (A.Chev.) A.Chev., Encycl. Biol. 22: 29 (1942). Type: ['Oubangui [Central African Republic]']. Type not designated.

Coffea aruwimiensis De Wild., Miss. Ém. Laurent 1: 321 (1906). *Coffea dewevrei* var. *aruwimiensis* (De Wild.) A.Chev., Encycl. Biol. 22: pl. 10 (1942). Types: [All Central African Republic] ['cultivé à Basoko, 21 i 1904, *E. and M. Laurent* s.n.; cultivé à Liranga (Ubangi), 27 I 1904, *E. and M. Laurent* s.n.; Wanie Rukula, 16 i 1904, *E. and M. Laurent* s.n.; Monga (Ubangi), 1905, sine coll.'] (syntypes BR).

Coffea royauxii De Wild., Miss. Ém. Laurent 1: 326 (1906). Type: [Central Africa Republic] ['Banzyville [Mobayi] (Ubangi), ii 1891, *L. Royaux* s.n.'] (syntypes BR!, K!)

Coffea liberica var. *dewevrei* forma *bwambensis* Bridson, Kew Bull. 37: 314 (1982). Type: Uganda, Toro District, Muntandi, Bwamba, *W.J. Eggeling* 3388 (holotype K!; isotype EA).

Illustrations: De Wildeman (1906: pl. 74 [as *C. arnoldiana*], pl. 75 [as *C. dewevrei*), pl. 78 [as *C. royauxii*], pl. 105 [as *C. dybowskii*])[46]; Chevalier (1929, fig. 13 [as *C. excelsa*)[19]; Lebrun (1941: pl. 15–19. excl. pl. 17, fig. 7 [as *C. liberica* var. *dewevrei*])[7]; Chevalier (1942: pl. 2–10)[15].

Literature: Cheney (1925: 93–95 [as *C. excelsa*])[47]; Chevalier (1942: 22, 29)[15]; Chevalier (1947: 180–186)[17]; Lebrun (1941 (168–175 [as *liberica* var. *dewevrei*])[26].

Distribution: Republic of the Congo, Cameroon, Democratic Republic of the Congo, Central African Republic, South Sudan, Uganda. Fig. 3.

Ecology: Humid, evergreen forest; (300–)440–770(−1825) m.

Name of uncertain affinity

Coffea zenkeri K.Krause ex De Wild., Ann. Jard. Bot. Buitenzorg, suppl. 3(1): 382 (1910). *Coffea dewevrei* var. *zenkeri* (K.Krause ex De Wild.) A.Chev., Encycl. Biol. 22: 29, pl. 13 (1942). Type: Not designated. Notes. Probably a synonym of *C. liberica*.

References

1 Pritchard, J.K., Stephens, M. & Donnelly, P. Inference of population structure using multilocus genotype data. *Genetics* **155**, 945–959 (2000).

2 Earl, D.A. & vonHoldt, B.M. STRUCTURE HARVESTER: a website and program for visualizing STRUCTURE output and implementing the Evanno method. *Conserv. Genet. Resour.* **4**, 359–361 (2012).

3 Evanno, G., Regnaut, S. & Goudet, J. Detecting the number of clusters of individuals using the software structure: a simulation study. *Mol. Ecol.* **14**, 2611–2620 (2005).

4 Hiern, W.P. On the African species of the genus *Coffea*. *Trans. Linn. Soc. London. 2nd Series. Botany*, **1,** 169–176 (1876).

5 Bull, W. *Coffea liberica. A retail list of new, beautiful and rare plants* (1874).

6 Bridson, D.M. The lectotypification of *Coffea liberica* (Rubiaceae). *Kew Bull.* **40**, 805–807 (1984).

7 Bridson, D.M. The lectotypification of *Coffea liberica* (Rubiaceae). *Kew Bull.* **40**, 805–807 (1985).

8 McCook, S. Ephemeral Plantations: The Rise and Fall of Liberian Coffee, 1870–1900. In: Comparing Apples, Oranges, and Cotton. Environmental Histories of the Plantation (ed. Uekötter, F.). Frankfurt/New York: Campus Verlag. 85–112 (2014).

9 Davis, A.P., Kiwuka, C., Faruk, A., Walubiri, M.J. & Kalema, J. The re-emergence of Liberica coffee as a major crop plant. *Nature Plants* **8**, 1322–1328 (2022).

10 Derry, R. Liberian coffee at the Straits Settlements (*Coffea liberica* Bull.). *Bull. Misc. Inform. (Royal Bot. Gard. Kew)* **23**, 261–263 (1888).

11 Cruwell, G.A. *Liberian Coffee In Ceylon: The History of the Introduction and Progress of the Cultivation up to April 1878* (A.M. & J. Ferguson 1878).

12 Durand, T. & De Wildeman, E. Matériaux pour la Flore du Congo. Sixième Fascicule. *Bull. Soc. Roy. Bot. Belgique* **38**, 171–220 (1900).

13 Davis, A.P., Govaerts, R., Bridson, D.M. & Stoffelen, P. An annotated taxonomic conspectus of the genus *Coffea* (Rubiaceae). *Bot. J. Linn. Soc.* **152**, 465–512 (2006).

14 Stoffelen, P. *Coffea and Psilanthus in tropical Africa: a systematic and palynological study, including a revision of the West and Central African species* (PhD thesis, Katholieke Universiteit Leuven 1998).

15 Chevalier, A. Les caféiers du globe, fasc. 2: Iconographie des caféiers sauvages et cultivés. *Ency. Biol.* **22**, 1–36, 158 plates (1942).

16 Chevalier, A. Nouveaux documents sur le Caféier Chari. *J. Agric. Trad. Bot. Appl.* **63**, 667–675 (1926).

17 Chevalier, A. Les caféiers du globe, fasc. 3: systématique des caféiers et faux-caféiers. Maladies et insectes nuisibles. *Ency. Biol.* **28**, 212 (1947).

18 De Wildeman, E. *Les Caféiers I.* (Veuve Monnom, 1901).

19 Chevalier, A. Les caféiers du globe, fasc. 1: Généralités sur les caféiers. *Ency. Biol.* **5**, 1–196 (1929).

20 Cramer, P.J.S. Gevens over de variabiliteit van de in Nederlandsch-Indië verbouwde koffie-sorten. *Meded. uitgaande van het Depart. Landbouw.* **1**, 1–696 (1913).

21 Cramer, P.J.S. *A Review of Literature of Coffee Research in Indonesia* (Turrialba: SIC Editorial, Inter-American Institute of Agricultural Sciences 1957).

22 Portères, R. Etude sur les caféiers spontanés de la section "Des Eucoffeae". Leur répartition, leur habitat, leure mise en culture et leur sélection en Cote d'Ivoire. Deuxième partie: Espèce, variétés et formes. *Annal. Agric. Afr. Occid.* **1**, 219–283 (1937).

23 Portères, R. Etude sur les caféiers spontanés de la section "Des Eucoffeae". Leur répartition, leur habitat, leure mise en culture et leur sélection en Cote d'Ivoire. Première partie: Répartition et habitat. *Annal. Agric. Afr. Occid.* **1**, 68–91 (1937).

24 Portères, R. Etude sur les caféiers spontanés de la section "Des Eucoffeae". Leur répartition, leur habitat, leure mise en culture et leur sélection en Cote d'Ivoire. Troisième partie: Mise en culture et sélection. *Annal. Agric. Afr. Occid.* **1**, 406–439 (1937).
25 Wrigley, G. *Coffee – Tropical Agriculture Series*. (Longman Scientific & Technical, 1988).
26 Lebrun, J. Recherches morphologiques et systématiques sur les caféiers du Congo. *Publications de L'institut National* **11**, 1–186 (1941).
27 Portères, R. Note botanique sur le *Coffea excelsa* A.Chev. (sensu lato) et le *Coffea macrochlamys* K.Schum. *Rev. Bot. Appl. Agric. Trop.* **16:**, 45–49 (1936).
28 Chevalier, A. Sur quelques Caféiers et Faux Caféiers de l'Angola et du Mayombe portugais. *Rev. Bot. Appl. Agric. Trop.* **19**, 396–407 (1939).
29 Bridson, D.M. in *Flora of Tropical East Africa, Rubiaceae, Part 2* (eds R.M. Polhill, D.M. Bridson, & B. Verdcourt) 703–723 (Balkema 1988).
30 N'Diaye, A.N., Poncet, V., Louran, J., Hamon, S. & Noirot, M. Genetic differentiation between *Coffea liberica* var. *liberica* and *C. liberica* var. *dewevrei* and comparison with *C. canephora*. *Plant Syst. Evol.* **253**, 95–104 (2005).
31 Baltazar, M.D. & Buot, I.E. Leaf architectural analysis of taxonomic confusing coffee species: *Coffea liberica* and *Coffea liberica* var. *dewevrei*. *Biodiversitas* **20**, 1560–1567 (2019).
32 Panaligan, A.C., Baltazar, M.D. & Alejandro, G.J.D. Molecular authentication of commercially cultivated coffee (*Coffea* spp.) in the Philippines using DNA barcodes. *Int. J. Agric. Biol.* **25**, 27–230 (2021).
33 Panaligan, A.C., Baltazar, M.D. & Alejandro, G.J.D. Genetic polymorphism of registered and popularly cultivated coffee (*Coffea* spp.) in the Philippines using inter-simple sequence repeats markers. *Biodiversitas* **21**, 4228–4233 (2020).
34 Hamon, P. *et al.* Genotyping-by-sequencing provides the first well-resolved phylogeny for coffee (*Coffea*) and insights into the evolution of caffeine content in its species. *Mol. Phylogenet. Evol.* **109**, 351–361 (2017).
35 Davis, A.P., Kiwuka, C., Faruk, A., Mulumba, J. & Kalema, J. A review of the indigenous coffee resources of Uganda and their potential for coffee sector sustainability and development. *Front. Pl. Sci.* **13**, 1057317 (2023).
36 Keay, R.W.J. *Coffea*. In: Hutchinson J, Dalziel JM, eds. *Flora of West Tropical Africa, 2nd edn, Vol. 2* (London: Crown Agents for Oversea Governments and Administrations 1963).
37 Mwanga, I.J.-C. & Stoffelen, P. in *Flore d'Afrique Centrale: Spermatophytes, Rubiaceae, Tribu VIII. Coffeeae* (ed E. Robbrecht) (Jardin Botanique de Meise 2024).
38 De Wildeman, E. *Mission Émile Laurent (1903–1904), Vol. 1 (Texte)*. (F. Vandenbuggenhoudt 1906).
39 Chevalier, A. Énumération des plantes récoltées. *Explor. Bot. Afrique Occ. Franç.* **1**, 334–337 (1920).
40 Chevalier, A. Les caféiers sauvages de la Guinée françiaise. Note de M.A.Chevalier, présentée par M. Ph. van Tieghem. *C. R. Hebd. Acad. Sci.* **140**, 1472–1475 (1905).
41 Turland, N. J. *et al. International Code of Nomenclature for algae, fungi, and plants (Shenzhen Code) adopted by the Nineteenth International Botanical Congress Shenzhen, China, July 2017*. (Glashütten: Koeltz Botanical Books 2018).
42 Davis, A. P. *et al.* The wild coffee resources of Uganda: a precious heritage. *Royal Botanic Gardens, Kew*, 1–44, doi:10.34885/fdtw-m431 (2023).
43 Krishnan, S., Ranker, T. A., Davis, A.P. & Rakotomalala, J.-J. An assessment of the genetic integrity of ex situ germplasm collections of three endangered species of

Coffea from Madagascar: implications for the management of field germplasm collections. *Genet. Resour. Crop Evol.* **60**, 1021–1036 (2013).
44 Zhang, J.J., Ye, Q.G., Yao, X.H. & Huang, H. W. Spontaneous interspecific hybridization and patterns of pollen dispersal in ex situ populations of a tree species (*Sinojackia xylocarpa*) that is extinct in the wild. *Conserv. Biol.* **24**, 246–255 (2009).
45 Suso, M., Gilsanz, S., Duc, G., Marget, P. & Moreno, M.T. Germplasm management of faba bean (*Vicia faba* L.): monitoring intercrossing between accessions with inter-plot barriers. *Gen. Resour. Crop Evol.* **53**, 1427–1439 (2006).
46 De Wildeman, E. *Mission Émile Laurent (1903–1904), Vol. 2 (Planches)*. (F. Vandenbuggenhoudt 1906).
47 Cheney, R. H. *A Monograph of the Economic Species of the Genus Coffea L*. (The New York University Press 1925).
