# Supplementary material for: Genomic data define species delimitation in Liberica coffee with implications for crop development and conservation
Source: Nat Plants. 2025 Aug 8;11(9):1729–38. doi: 10.1038/s41477-025-02073-y (PMC12449269; doi:10.1038/s41477-025-02073-y)
Supplement: Supplementary file 3 — Supplementary Table 1. List of samples with key accession data and sequencing details. Supplementary Table 2. t-test results for morphological data. Supplementary Table 3. Summary of Bioclim data and elevation for C. liberica, C. dewevrei and C. klainei. Supplementary Table 4. t-test results for Bioclim data for C. liberica, C. dewevrei and C. klainei. Supplementary Table 5. European Nucleotide Archive ID and file codes. [file 41477_2025_2073_MOESM3_ESM.pdf]

Supplementary Table 1. List of samples with key accession data and sequencing details. Material type: H = Herbarium specimen (K); S(comm) = seed from commercial sources; S (EBC) = Seed (Economic Botany Collection, RBG, Kew); S(H) = seed from herbarium samples; SDL = silica gel dried leaf tissue

Species	Collector name and number	Material type	Year of collection	Country	Cult. (C) or wild (W)	DNA Library code	No. of quality-filtered paired reads	No. of reads on target	Enrichment efficiency (% reads on target)	No. of genes assembled at 50%	Total bp recovered	Efficiency recovery (% bp recovered compared to reference)
C. bakossii Cheek & Bridson	Musah 97	H	1995	Cameroon	W	23H69	4947039	2162644	0.437	324	249465	86.7
C. bakossii Cheek & Bridson	Sonké 4590	H	1998	Cameroon	W	23H70	4075231	1793583	0.44	322	241374	83.9
C. brevipes Hiern	Maurin OM20	H	2002	Cameroon	W	23C81	2546482	537101	0.211	317	244128	84.9
C. canephora Pierre ex A.Froehner	Samuel 3105	H	2010	Sri Lanka	C	23C03	4024704	2635800	0.655	327	254685	88.5
C. dewevrei De Wild. & T.Durand	Haggar 14-16	SDL	2017	Sierra Leone	C	23F66	4418859	2272340	0.514	333	260361	90.5
C. dewevrei De Wild. & T.Durand	Haggar 16-16	SDL	2017	Sierra Leone	C	23F67	4672601	2618651	0.56	335	262188	91.2
C. dewevrei De Wild. & T.Durand	Haggar 18-16	SDL	2017	Sierra Leone	C	23F68	5964078	2997985	0.503	325	253644	88.2
C. dewevrei De Wild. & T.Durand	Billiet BR 19370062	SDL	2002	DRC	W	23D65	6506279	3086845	0.474	329	257184	89.4
C. dewevrei De Wild. & T.Durand	BR 2174	SDL	2002	DRC	W	23D44	4760666	2288194	0.481	328	258123	89.7
C. dewevrei De Wild. & T.Durand	CEPAH	SDL	2017	Sierra Leone	C	23F63	3227875	1192457	0.369	311	238062	82.8
C. dewevrei De Wild. & T.Durand	Couch A124	H	2022	Guinea C.	C	23D51	4442864	2170879	0.489	324	257190	89.4
C. dewevrei De Wild. & T.Durand	Fay 5187	H	1982	CAR	W	23C18	4499263	2974267	0.661	319	245724	85.4
C. dewevrei De Wild. & T.Durand	Friis & Vollesen 1981	H	1980	South Sudan	W	23H28	5420518	2044596	0.377	320	241479	84.0
C. dewevrei De Wild. & T.Durand	Hernandez 3692	S(comm)	2019	Costa Rica	C	23D49	3496402	1846425	0.528	320	247506	86.0
C. dewevrei De Wild. & T.Durand	IRD EB53	SDL	2001	CAR	W	23D66	5390570	2258127	0.419	331	259059	90.1
C. dewevrei De Wild. & T.Durand	IRD EB60	SDL	2001	CAR	W	23D68	8841928	3977756	0.45	328	259155	90.1
C. dewevrei De Wild. & T.Durand	Kawabata A10	S(comm)	2019	Hawaii	C	23D59	2482072	1103466	0.445	323	251331	87.4
C. dewevrei De Wild. & T.Durand	Kawabata A9	S(comm)	2019	Hawaii	C	23D58	3066885	1329352	0.433	324	254562	88.5
C. dewevrei De Wild. & T.Durand	Liengola 130	H	1996	DRC	W	23H78	2556379	1307975	0.512	304	233463	81.2
C. dewevrei De Wild. & T.Durand	Sarmu SL 4	S(comm)	2019	Sierra Leone	C	23D50	3698944	1773361	0.479	322	251262	87.4
C. dewevrei De Wild. & T.Durand	Slow Foods	S(comm)	2016	Uganda	C	23C04	3950162	2404736	0.609	328	256209	89.1
C. dewevrei De Wild. & T.Durand	Slow Foods	S(comm)	2016	Uganda	C	23C05	4534466	2726510	0.601	329	258393	89.8
C. dewevrei De Wild. & T.Durand	Snowden 1697	H	1930	South Sudan	W	23D63	7565236	3767361	0.498	318	248862	86.5
C. dewevrei De Wild. & T.Durand	Sonké 3096	H	2003	CAR	W	23C14	884188	460122	0.52	280	208305	72.4
C. dewevrei De Wild. & T.Durand	Thomas 4026	H	1941	Uganda	W	23H26	5194226	2677659	0.516	332	262125	91.1
C. dewevrei De Wild. & T.Durand	Sarmu SL 3	S(comm)	2019	Sierra Leone	C	23C02	3626288	2229106	0.615	327	258090	89.7
C. eugenioides S.Moore	Harley 9332	H	1955	Tanzania	W	23G93	5705525	3177563	0.557	333	260157	90.4
C. humilis A.Chev.	IRD G56	SDL	2002	Ivory Coast	W	23C94	3876086	2292006	0.591	326	253968	88.3
C. klainei Pierre ex De Wild.	Jongkind 8693	H	1975	Cameroon	W	23C08	2857669	1822280	0.638	320	247509	86.0
C. klainei Pierre ex De Wild.	McPherson 16219	H	1993	Gabon	W	23C12	5225488	3004300	0.575	322	253236	88.0
C. klainei Pierre ex De Wild.	Tchiengue 3740	H	2016	Cameroon	W	23C19	6171919	3571994	0.579	326	254361	88.4
C. leonimontana Stoff.	Mbatchou 399	H	1992	Cameroon	W	23H68	1990456	947852	0.476	301	222429	77.3
C. leonimontana Stoff.	Osborne 225	H	2006	Cameroon	W	23F48	2053486	1277880	0.622	270	197682	68.7
C. liberica W.Bull	Davis s.n.	S(comm)	2023	Madagascar	C	23H72	3483084	1442817	0.414	330	258306	89.8
C. liberica W.Bull	De Wilde 156	H	1963	Ivory Coast	C	23C17	4536028	2569405	0.566	323	252333	87.7
C. liberica W.Bull	Dr Trimen s.n.	S(EBC)	1892	Sri Lanka	C	23H84	5111480	3382357	0.662	326	247659	86.1
C. liberica W.Bull	Enti + Jenik 36571	H	1967	Ghana	W	23C13	2609196	1101309	0.422	305	228147	79.3
C. liberica W.Bull	House of Kendal A125	S©	2022	Malaysia	C	23D52	3435089	1698992	0.495	327	254184	88.4
C. liberica W.Bull	IRD EA 67	SDL	2002	Ivory Coast	W	23D46	4646017	2479424	0.534	329	259539	90.2
C. liberica W.Bull	Jones & Onochie 17214	H	1946	Nigeria	W	23C16	2621740	1762567	0.672	313	235659	81.9
C. liberica W.Bull	Millen s.n.	S(EBC)	1895	Nigeria	C	23H85	3884242	2426939	0.625	318	239517	83.3
C. liberica W.Bull	Millen s.n.	S(H)	1895	Nigeria	C	23H79	3305171	1635502	0.495	326	252288	87.7
C. liberica W.Bull	Swaray 213	H	2013	Sierra Leone	W	23H29	8851599	3910987	0.442	331	252585	87.8
C. liberica W.Bull	My Liberica	S(comm)	2023	Malaysia	C	24L80	2948467	514810	0.174	314	236412	82.2
C. magnistipula Stoff. & Robbr.	Bretler 8155	S(H)	1986	Gabon	W	23F54	3222070	1319919	0.409	45	56523	19.7
C. mapiana Sonké, Nguembou & A.P.Davis	Sonké 3694	H	2005	Cameroon	W	23G96	4111266	1293199	0.315	333	261408	90.9
C. mapiana Sonké, Nguembou & A.P.Davis	Sonké 3826	H	2005	Cameroon	W	23H25	4289957	2131041	0.497	326	255333	88.8
C. mannii (Hook.f)A.P.Davis	Maurin OM1	H	2002	Cameroon	W	23D70	5838238	2466638	0.422	321	252867	87.9
C. montekupensis Stoff.	Davis APD3010	H	2002	Cameroon	W	23D73	2432004	928512	0.382	324	252381	87.7
C. montekupensis Stoff.	Maurin 10	SDL	2002	Cameroon	W	24L61	11512878	3262593	0.283	332	258549	89.9
C. montekupensis Stoff.	Tchiengue B.2617	H	2006	Cameroon	W	24L62	9172394	3628002	0.395	325	250878	87.2
C. rizetiana Stoff. & Noirot	IRD EA 67 [a]	SDL	2002	Cameroon	W*	24L55	7913562	3379514	0.427	325	253659	88.2
C. rizetiana Stoff. & Noirot	IRD EA 67 [b]	SDL	2002	Cameroon	W*	19C86	5995190	374995	0.063	283	196392	75.3
C. stenophylla G.Don.	Daniell s.n.	S(EBC)	1873	Sierra Leone	C	23F71	5399422	3376714	0.625	329	258447	89.9

Supplementary Table 2a. *t*-Test results for morphological data. See main text (Table 1), and Supplementary Table 2b for details. *t*-Test: Two sample assuming unequal variance.

Character/variable	Species		Species	*p*-values	Significance
Leaf length (cm)	*C. liberica*	vs.	*C. dewevrei*	1.52391E-10	p < 0.0005
	C. liberica	vs.	*C. klainei*	0.393089164	
	C. dewevrei	vs.	*C. klainei*	0.000175777	p < 0.0005
Leaf width (cm)	*C. liberica*	vs.	*C. dewevrei*	6.82338E-22	p < 0.0005
	C. liberica	vs.	*C. klainei*	0.38479848	
	C. dewevrei	vs.	*C. klainei*	3.97113E-12	p < 0.0005
Seed length (mm)	*C. liberica*	vs.	*C. dewevrei*	3.13501E-43	p < 0.0005
Seed width (mm)	*C. liberica*	vs.	*C. dewevrei*	2.04131E-37	p < 0.0005
Parchment thickness (mm)	*C. liberica*	vs.	*C. dewevrei*	4.39867E-24	p < 0.0005
Elevation (mm)	*C. liberica*	vs.	*C. dewevrei*	0.000407548	p < 0.0005
	C. liberica	vs.	*C. klainei*	0.124916587	
	C. dewevrei	vs.	*C. klainei*	8.69976E-09	p < 0.0005

Supplementary Table 2b. N number (n =) and quartile values (see Extended Data Fig. 1).

Character/variable	Species	n =	Q0 (min)	Q1	Q2 (median)	Q3	Q4 (max)
Leaf length (cm)	*C. liberica*	32	16.7	19.8	23.3	24.8	30.2
	C. dewevrei	36	22.4	26.7	28.3	31.9	35.9
	C. klainei	26	15	17.7	22.2	30.3	33.6
Leaf width (cm)	*C. liberica*	32	5.6	7.4	7.6	9	10.4
	C. dewevrei	36	10	13	13.5	14.5	18
	C. klainei	26	5.5	6.4	8.2	10.8	14.4
Seed length (mm)	*C. liberica*	158	9.5	11.5	12.3	13.3	18.3
	C. dewevrei	61	7.7	8.6	9.4	10	11.3
Seed width (mm)	*C. liberica*	158	6.5	7.8	8.3	8.8	12
	C. dewevrei	61	5.4	6.2	6.6	6.9	8
Parchment thickness (mm)	*C. liberica*	101	0.36	0.51	0.59	0.63	0.77
	C. dewevrei	21	0.22	0.27	0.32	0.35	0.41
Elevation (m)	*C. liberica*	19	130	215	375	502	785
	C. dewevrei	267	305	437	628	767	1824
	C. klainei	25	13	60	296	438	585

Supplementary Table 3. Summary of Bioclim data and elevation (m) for *C. liberica*, *C. dewevrei* and C. *klainei*. See Supplementary Table 4 for further information. Box and whisker plots are given in Supplementary Fig. 4.

Species		bio1	bio10	bio11	bio12	bio13	bio14	bio15	bio16	bio17
C. liberica	Min.	22.8	24.3	21.5	1773.4	280.4	8.0	47.6	697.1	35.1
	**Mean**	**24.6**	**26.2**	**23.2**	**2215.3**	**376.6**	**15.9**	**66.9**	**988.9**	**80.0**
	Max.	25.7	27.1	24.2	3115.3	605.1	39.9	84.3	1580.0	177.7
C. klainei	Min.	23.3	24.4	21.1	1441.3	246.0	2.7	48.6	605.2	14.5
	**Mean**	**24.4**	**25.5**	**23.0**	**2062.0**	**355.1**	**19.2**	**64.5**	**902.2**	**97.8**
	Max.	25.7	26.7	24.7	3366.4	608.4	120.6	73.1	1480.6	418.5
C. dewevrei	Min.	17.8	18.3	17.5	983.4	142.1	0.3	19.6	389.5	1.8
	**Mean**	**24.4**	**25.7**	**23.4**	**1678.0**	**230.0**	**36.2**	**49.3**	**643.8**	**141.1**
	Max.	27.1	29.7	25.5	4069.6	498.2	175.0	98.7	1383.7	620.2
**Species**		**bio1**	**bio10**	**bio11**	**bio12**	**bio13**	**bio14**	**bio15**	**bio16**	**bio17**
C. liberica	Min.	22.8	24.3	21.5	1773.4	280.4	8.0	47.6	697.1	35.1
C. klainei	Min.	23.3	24.4	21.1	1441.3	246.0	2.7	48.6	605.2	14.5
C. dewevrei	Min.	17.8	18.3	17.5	983.4	142.1	0.3	19.6	389.5	1.8
C. liberica	**Mean**	**24.6**	**26.2**	**23.2**	**2215.3**	**376.6**	**15.9**	**66.9**	**988.9**	**80.0**
C. klainei	**Mean**	**24.4**	**25.5**	**23.0**	**2062.0**	**355.1**	**19.2**	**64.5**	**902.2**	**97.8**
C. dewevrei	**Mean**	**24.4**	**25.7**	**23.4**	**1678.0**	**230.0**	**36.2**	**49.3**	**643.8**	**141.1**
C. liberica	Max.	25.7	27.1	24.2	3115.3	605.1	39.9	84.3	1580.0	177.7
C. klainei	Max.	25.7	26.7	24.7	3366.4	608.4	120.6	73.1	1480.6	418.5
C. dewevrei	Max.	27.1	29.7	25.5	4069.6	498.2	175.0	98.7	1383.7	620.2

Species		bio18	bio19	bio2	bio3	bio4	bio5	bio6	bio7	bio8	bio9	elev
C. liberica	Min.	199.9	483.6	7.2	0.6	63.3	29.7	17.2	9.2	21.5	23.5	130
	**Mean**	**311.6**	**961.7**	**7.9**	**0.7**	**110.3**	**32.2**	**20.1**	**12.1**	**23.6**	**25.2**	**386**
	Max.	445.5	1580.0	8.9	0.8	150.6	33.9	21.8	15.1	25.0	26.6	785
C. klainei	Min.	396.8	14.5	4.2	0.6	54.9	28.9	17.0	6.5	23.1	21.1	13
	**Mean**	**643.2**	**146.7**	**6.8**	**0.7**	**98.7**	**29.7**	**19.7**	**10.0**	**24.6**	**23.2**	**274**
	Max.	975.3	726.4	8.2	0.9	156.7	31.2	22.7	12.3	25.7	25.9	585
C. dewevrei	Min.	42.7	8.7	5.2	0.6	28.1	23.1	12.9	6.9	17.6	17.8	305
	**Mean**	**273.2**	**597.7**	**8.9**	**0.7**	**93.4**	**32.2**	**19.7**	**12.5**	**23.4**	**25.1**	**654**
	Max.	1012.8	1332.8	10.9	0.9	180.2	36.9	23.3	16.8	25.7	27.9	1824
**Species**		**bio18**	**bio19**	**bio2**	**bio3**	**bio4**	**bio5**	**bio6**	**bio7**	**bio8**	**bio9**	**elev**
C. liberica	Min.	199.9	483.6	7.2	0.6	63.3	29.7	17.2	9.2	21.5	23.5	130
C. klainei	Min.	396.8	14.5	4.2	0.6	54.9	28.9	17.0	6.5	23.1	21.1	13
C. dewevrei	Min.	42.7	8.7	5.2	0.6	28.1	23.1	12.9	6.9	17.6	17.8	305
C. liberica	**Mean**	**311.6**	**961.7**	**7.9**	**0.7**	**110.3**	**32.2**	**20.1**	**12.1**	**23.6**	**25.2**	**386**
C. klainei	**Mean**	**643.2**	**146.7**	**6.8**	**0.7**	**98.7**	**29.7**	**19.7**	**10.0**	**24.6**	**23.2**	**274**
C. dewevrei	**Mean**	**273.2**	**597.7**	**8.9**	**0.7**	**93.4**	**32.2**	**19.7**	**12.5**	**23.4**	**25.1**	**654**
C. liberica	Max.	445.5	1580.0	8.9	0.8	150.6	33.9	21.8	15.1	25.0	26.6	785
C. klainei	Max.	975.3	726.4	8.2	0.9	156.7	31.2	22.7	12.3	25.7	25.9	585
C. dewevrei	Max.	1012.8	1332.8	10.9	0.9	180.2	36.9	23.3	16.8	25.7	27.9	1824

BIO1 = Annual Mean Temperature
BIO2 = Mean Diurnal Range (Mean of monthly (max temp - min temp))
BIO3 = Isothermality (BIO2/BIO7) (×100)
BIO4 = Temperature Seasonality (standard deviation ×100)
BIO5 = Max Temperature of Warmest Month
BIO6 = Min Temperature of Coldest Month
BIO7 = Temperature Annual Range (BIO5-BIO6)
BIO8 = Mean Temperature of Wettest Quarter
BIO9 = Mean Temperature of Driest Quarter
BIO10 = Mean Temperature of Warmest Quarter
BIO11 = Mean Temperature of Coldest Quarter
BIO12 = Annual Precipitation
BIO13 = Precipitation of Wettest Month
BIO14 = Precipitation of Driest Month
BIO15 = Precipitation Seasonality (Coefficient of Variation)
BIO16 = Precipitation of Wettest Quarter
BIO17 = Precipitation of Driest Quarter
BIO18 = Precipitation of Warmest Quarter
BIO19 = Precipitation of Coldest Quarter
Elevation (m)

Supplementary Information Table 4. *t*-Test results for Bioclim data for *C. liberica*, *C. dewevrei* and *C. klainei*. See Supplementary Table 3 for further information. *t*-Test: two sample assuming unequal variance. N numbers (n =) are provided in the main text. Box and whisker plots are given in Supplementary Fig. 4.

Character/variable	Species	Vs.	Species	*p*-values	Significance
BIO1 = Annual Mean Temperature	*C. liberica*	vs.	*C. dewevrei*	0.422317478	
	C. liberica	vs.	*C. klainei*	0.549194336	
	C. dewevrei	vs.	*C. klainei*	0.869665652	
BIO2 = Mean Diurnal Range (Mean of monthly (max temp - min temp))	*C. liberica*	vs.	*C. dewevrei*	8.11113E-06	$p < 0.0005$
	C. liberica	vs.	*C. klainei*	0.003326598	$p < 0.005$
	C. dewevrei	vs.	*C. klainei*	3.15885E-07	$p < 0.0005$
BIO3 = Isothermality (BIO2/BIO7) (×100)	*C. liberica*	vs.	*C. dewevrei*	0.000524103	$p < 0.005$
	C. liberica	vs.	*C. klainei*	0.176217645	
	C. dewevrei	vs.	*C. klainei*	0.220370569	
BIO4 = Temperature Seasonality (standard deviation ×100)	*C. liberica*	vs.	*C. dewevrei*	0.025745339	$p < 0.05$
	C. liberica	vs.	*C. klainei*	0.274248209	
	C. dewevrei	vs.	*C. klainei*	0.576176416	
BIO5 = Max Temperature of Warmest Month	*C. liberica*	vs.	*C. dewevrei*	0.996843858	
	C. liberica	vs.	*C. klainei*	2.54563E-06	$p < 0.0005$
	C. dewevrei	vs.	*C. klainei*	3.62337E-15	$p < 0.0005$
BIO6 = Min Temperature of Coldest Month	*C. liberica*	vs.	*C. dewevrei*	0.375416275	
	C. liberica	vs.	*C. klainei*	0.511050067	
	C. dewevrei	vs.	*C. klainei*	0.984748332	
BIO7 = Temperature Annual Range (BIO5-BIO6)	*C. liberica*	vs.	*C. dewevrei*	0.415999721	
	C. liberica	vs.	*C. klainei*	0.001489099	$p < 0.005$
	C. dewevrei	vs.	*C. klainei*	8.47628E-06	$p < 0.0005$
BIO8 = Mean Temperature of Wettest Quarter	*C. liberica*	vs.	*C. dewevrei*	0.587819428	
	C. liberica	vs.	*C. klainei*	0.00692304	$p < 0.05$
	C. dewevrei	vs.	*C. klainei*	9.8663E-06	$p < 0.0005$
BIO9 = Mean Temperature of Driest Quarter	*C. liberica*	vs.	*C. dewevrei*	0.701018508	
	C. liberica	vs.	*C. klainei*	9.03934E-05	$p < 0.0005$
	C. dewevrei	vs.	*C. klainei*	3.89231E-05	$p < 0.0005$
BIO10 = Mean Temperature of Warmest Quarter	*C. liberica*	vs.	*C. dewevrei*	0.150110729	
	C. liberica	vs.	*C. klainei*	0.039340965	$p < 0.05$
	C. dewevrei	vs.	*C. klainei*	0.442216841	
BIO11 = Mean Temperature of Coldest Quarter	*C. liberica*	vs.	*C. dewevrei*	0.676956934	
	C. liberica	vs.	*C. klainei*	0.567940721	
	C. dewevrei	vs.	*C. klainei*	0.313623664	
BIO12 = Annual Precipitation	*C. liberica*	vs.	*C. dewevrei*	0.003444747	$p < 0.005$
	C. liberica	vs.	*C. klainei*	0.466471561	
	C. dewevrei	vs.	*C. klainei*	0.020363958	$p < 0.05$
BIO13 = Precipitation of Wettest Month	*C. liberica*	vs.	*C. dewevrei*	0.000728913	$p < 0.005$
	C. liberica	vs.	*C. klainei*	0.618056199	
	C. dewevrei	vs.	*C. klainei*	0.000201795	$p < 0.0005$
	C. liberica	vs.	*C. dewevrei*	1.75558E-05	$p < 0.0005$

BIO14 = Precipitation of Driest Month	*C. liberica*	vs.	*C. klainei*	0.631014689	
	C. dewevrei	vs.	*C. klainei*	0.017287129	$p < 0.05$
BIO15 = Precipitation Seasonality (Coefficient of Variation)	*C. liberica*	vs.	*C. dewevrei*	2.07183E-08	$p < 0.0005$
	C. liberica	vs.	*C. klainei*	0.499344534	
	C. dewevrei	vs.	*C. klainei*	2.07183E-08	$p < 0.0005$
BIO16 = Precipitation of Wettest Quarter	*C. liberica*	vs.	*C. dewevrei*	0.002684878	$p < 0.005$
	C. liberica	vs.	*C. klainei*	0.448282099	
	C. dewevrei	vs.	*C. klainei*	0.00085748	$p < 0.005$
BIO17 = Precipitation of Driest Quarter	*C. liberica*	vs.	*C. dewevrei*	0.00057884	$p < 0.005$
	C. liberica	vs.	*C. klainei*	0.47377745	
	C. dewevrei	vs.	*C. klainei*	0.081702935	
BIO18 = Precipitation of Warmest Quarter	*C. liberica*	vs.	*C. dewevrei*	0.162217762	
	C. liberica	vs.	*C. klainei*	2.90302E-08	$p < 0.0005$
	C. dewevrei	vs.	*C. klainei*	2.32726E-09	$p < 0.0005$
BIO19 = Precipitation of Coldest Quarter	*C. liberica*	vs.	*C. dewevrei*	0.003102961	$p < 0.005$
	C. liberica	vs.	*C. klainei*	9.66833E-07	$p < 0.0005$
	C. dewevrei	vs.	*C. klainei*	4.21209E-11	$p < 0.0005$

BIO1 = Annual Mean Temperature
BIO2 = Mean Diurnal Range (Mean of monthly (max temp - min temp))
BIO3 = Isothermality (BIO2/BIO7) (×100)
BIO4 = Temperature Seasonality (standard deviation ×100)
BIO5 = Max Temperature of Warmest Month
BIO6 = Min Temperature of Coldest Month
BIO7 = Temperature Annual Range (BIO5-BIO6)
BIO8 = Mean Temperature of Wettest Quarter
BIO9 = Mean Temperature of Driest Quarter
BIO10 = Mean Temperature of Warmest Quarter
BIO11 = Mean Temperature of Coldest Quarter
BIO12 = Annual Precipitation
BIO13 = Precipitation of Wettest Month
BIO14 = Precipitation of Driest Month
BIO15 = Precipitation Seasonality (Coefficient of Variation)
BIO16 = Precipitation of Wettest Quarter
BIO17 = Precipitation of Driest Quarter
BIO18 = Precipitation of Warmest Quarter
BIO19 = Precipitation of Coldest Quarter

Supplementary Table 5. European Nucleotide Archive ID and file codes. The DNA library codes are used as the identifier: accession details are provided in Supplementary Table 1. Raw reads for Angiosperms353 sequence data are available at the European Nucleotide Archive (https://www.ebi.ac.uk) under project no. PRJEB78707.

Species	ENA ID code	File name	DNA Library code
C. bakossii	ERR13479610	23H69_2.fastq.gz	23H69
C. bakossii	ERR13479610	23H69_1.fastq.gz	23H69
C. bakossii	ERS22863148	23H70_1.fastq.gz	23H70
C. bakossii	ERS22863148	23H70_2.fastq.gz	23H70
C. brevipes	ERR13479608	23C81_2.fastq.gz	23C81
C. brevipes	ERR13479608	23C81_1.fastq.gz	23C81
C. canephora	ERR13479605	23C03_2.fastq.gz	23C03
C. canephora	ERR13479605	23C03_1.fastq.gz	23C03
C. dewevrei	ERR13479603	23F66_2.fastq.gz	23F66
C. dewevrei	ERR13479603	23F66_1.fastq.gz	23F66
C. dewevrei	ERR13479600	23F67_2.fastq.gz	23F67
C. dewevrei	ERR13479600	23F67_1.fastq.gz	23F67
C. dewevrei	ERR13479598	23F68_2.fastq.gz	23F68
C. dewevrei	ERR13479598	23F68_1.fastq.gz	23F68
C. dewevrei	ERR13479594	23D65_2.fastq.gz	23D65
C. dewevrei	ERR13479594	23D65_1.fastq.gz	23D65
C. dewevrei	ERR13479589	23D44_2.fastq.gz	23D44
C. dewevrei	ERR13479589	23D44_1.fastq.gz	23D44
C. dewevrei	ERR13479587	23F63_2.fastq.gz	23F63
C. dewevrei	ERR13479587	23F63_1.fastq.gz	23F63
C. dewevrei	ERR13479586	23D51_2.fastq.gz	23D51
C. dewevrei	ERR13479586	23D51_1.fastq.gz	23D51
C. dewevrei	ERR13479578	23C18_2.fastq.gz	23C18
C. dewevrei	ERR13479578	23C18_1.fastq.gz	23C18
C. dewevrei	ERR13479566	23H28_2.fastq.gz	23H28
C. dewevrei	ERR13479566	23H28_1.fastq.gz	23H28
C. dewevrei	ERR13479553	23D49_2.fastq.gz	23D49
C. dewevrei	ERR13479553	23D49_1.fastq.gz	23D49
C. dewevrei	ERR13479537	23D66_2.fastq.gz	23D66
C. dewevrei	ERR13479537	23D66_1.fastq.gz	23D66
C. dewevrei	ERR13479524	23D68_2.fastq.gz	23D68
C. dewevrei	ERR13479524	23D68_1.fastq.gz	23D68
C. dewevrei	ERR13479507	23D59_2.fastq.gz	23D59
C. dewevrei	ERR13479507	23D59_1.fastq.gz	23D59
C. dewevrei	ERR13479502	23D58_2.fastq.gz	23D58
C. dewevrei	ERR13479502	23D58_1.fastq.gz	23D58
C. dewevrei	ERR13479495	23H78_2.fastq.gz	23H78
C. dewevrei	ERR13479495	23H78_1.fastq.gz	23H78
C. dewevrei	ERR13479491	23D50_2.fastq.gz	23D50
C. dewevrei	ERR13479491	23D50_1.fastq.gz	23D50
C. dewevrei	ERR13479483	23C04_2.fastq.gz	23C04
C. dewevrei	ERR13479483	23C04_1.fastq.gz	23C04
C. dewevrei	ERR13476975	23C05_2.fastq.gz	23C05
C. dewevrei	ERR13476975	23C05_1.fastq.gz	23C05
C. dewevrei	ERR13476967	23D63_2.fastq.gz	23D63
C. dewevrei	ERR13476967	23D63_1.fastq.gz	23D63
C. dewevrei	ERR13476956	23C14_2.fastq.gz	23C14
C. dewevrei	ERR13476956	23C14_1.fastq.gz	23C14
C. dewevrei	ERR13476954	23H26_2.fastq.gz	23H26

C. dewevrei	ERR13476954	23H26_1.fastq.gz	23H26
C. eugenioides	ERR13476945	23G93_2.fastq.gz	23G93
C. eugenioides	ERR13476945	23G93_1.fastq.gz	23G93
C. humilis	ERR13476935	23C94_2.fastq.gz	23C94
C. humilis	ERR13476935	23C94_1.fastq.gz	23C94
C. klainei	ERR13476929	23C08_2.fastq.gz	23C08
C. klainei	ERR13476929	23C08_1.fastq.gz	23C08
C. klainei	ERR13476924	23C12_2.fastq.gz	23C12
C. klainei	ERR13476924	23C12_1.fastq.gz	23C12
C. klainei	ERR13476917	23C19_2.fastq.gz	23C19
C. klainei	ERR13476917	23C19_1.fastq.gz	23C19
C. leonimontana	ERR13476905	23H68_2.fastq.gz	23H68
C. leonimontana	ERR13476905	23H68_1.fastq.gz	23H68
C. leonimontana	ERR13476901	23F48_2.fastq.gz	23F48
C. leonimontana	ERR13476901	23F48_1.fastq.gz	23F48
C. liberica	ERS22863149	24L80_1.fastq.gz	24L80
C. liberica	ERS22863149	24L80_2.fastq.gz	24L80
C. liberica	ERR13476897	23H71_2.fastq.gz	23H71
C. liberica	ERR13476897	23H71_1.fastq.gz	23H71
C. liberica	ERR13476890	23H72_2.fastq.gz	23H72
C. liberica	ERR13476890	23H72_1.fastq.gz	23H72
C. liberica	ERR13476884	23C17_2.fastq.gz	23C17
C. liberica	ERR13476884	23C17_1.fastq.gz	23C17
C. liberica	ERR13476875	23H84_2.fastq.gz	23H84
C. liberica	ERR13476875	23H84_1.fastq.gz	23H84
C. liberica	ERR13476862	23C13_2.fastq.gz	23C13
C. liberica	ERR13476862	23C13_1.fastq.gz	23C13
C. liberica	ERR13476852	23D52_2.fastq.gz	23D52
C. liberica	ERR13476852	23D52_1.fastq.gz	23D52
C. liberica	ERR13476851	23D46_2.fastq.gz	23D46
C. liberica	ERR13476851	23D46_1.fastq.gz	23D46
C. liberica	ERR13476848	23C16_2.fastq.gz	23C16
C. liberica	ERR13476848	23C16_1.fastq.gz	23C16
C. liberica	ERR13476847	23H85_2.fastq.gz	23H85
C. liberica	ERR13476847	23H85_1.fastq.gz	23H85
C. liberica	ERR13476846	23H79_2.fastq.gz	23H79
C. liberica	ERR13476846	23H79_1.fastq.gz	23H79
C. dewevrei	ERR13476843	23C02_2.fastq.gz	23C02
C. dewevrei	ERR13476843	23C02_1.fastq.gz	23C02
C. liberica	ERR13476841	23H29_2.fastq.gz	23H29
C. liberica	ERR13476841	23H29_1.fastq.gz	23H29
C. mannii	ERR13476589	23D70_2.fastq.gz	23D70
C. mannii	ERR13476589	23D70_1.fastq.gz	23D70
C. mapiana	ERR13476233	23H25_2.fastq.gz	23H25
C. mapiana	ERR13476233	23H25_1.fastq.gz	23H25
C. mapiana	ERR13476591	23G96_2.fastq.gz	23G96
C. mapiana	ERR13476591	23G96_1.fastq.gz	23G96
C. montekupensis	ERR13476231	23D73_2.fastq.gz	23D73
C. montekupensis	ERR13476231	23D73_1.fastq.gz	23D73
C. montekupensis	ERS22863150	24L61_1.fastq.gz	24L61
C. montekupensis	ERS22863150	24L61_2.fastq.gz	24L61
C. montekupensis	ERS22863151	24L62_1.fastq.gz	24L62
C. montekupensis	ERS22863151	24L62_2.fastq.gz	24L62
C. rizetiana	ERS22863153	C86_1.fastq.gz	19C86
C. rizetiana	ERS22863153	C86_2.fastq.gz	19C86
C. rizetiana	ERS22863152	24L55_1.fastq.gz	24L55
C. rizetiana	ERS22863152	24L55_2.fastq.gz	24L55
C. stenophylla	ERR13474210	23F71_2.fastq.gz	23F71
C. stenophylla	ERR13474210	23F71_1.fastq.gz	23F71
